# Lactic acid promotes metastasis of papillary thyroid carcinoma by enhancing CPT1A lactylation

**DOI:** 10.1038/s41419-026-08790-2

**Published:** 2026-04-27

**Authors:** Mingjian Zhao, Haifeng Han, Min Sun, Ruowen Li, Huimin Chen, Fangyu Liu, Chengxu Miao, Yongkang Wu, Xiaojia Shi, Mengting Wu, Gabriele Materazzi, Paolo Miccoli, Jinghui Lu, Xuetian Yue

**Affiliations:** 1 Department of General Surgery, Qilu Hospital, Cheeloo College of Medicine, Shandong University, Jinan, 250012 China; 2 Department of General Surgery, Nanjing Jinling Hospital, Affiliated Hospital of Medical School, Nanjing University, Nanjing, 210002 China; 3 The Ninth Medical Center of the Chinese PLA General Hospital, Beijing, 100020 China; 4 Department of Cell Biology, School of Basic Medical Sciences, Cheeloo Medical College of Medicine, Shandong University, Jinan, 250012 China; 5 Department of Surgical, Medical, and Molecular Pathology and Critical Area, University of Pisa, 56124 Pisa, Italy

**Keywords:** Thyroid cancer, Cancer metabolism

## Abstract

Papillary thyroid carcinoma (PTC), the most prevalent thyroid malignancy, exhibits aggressive behavior in subsets with metastasis. Despite advances in risk stratification, biomarkers predicting metastatic potential remain limited. Here, we identify lactate as a critical driver of PTC metastasis through lactylation of carnitine palmitoyltransferase 1 A (CPT1A), the rate-limiting enzyme in fatty acid β-oxidation (FAO). Multi-omics profiling of 27 paired PTC tissues revealed elevated lactate levels and FAO activation, corroborated by TCGA data. Functional assays demonstrated that exogenous lactate enhances PTC cell migration via CPT1A-dependent FAO. Mechanistically, lactate upregulated CPT1A transcription by promoting histone H3K18 lactylation (H3K18la), simultaneously stabilized CPT1A protein via lactylation of CPT1A at K180/K285 to suppress its ubiquitin-proteasomal degradation. Genetic or pharmacological inhibition of CPT1A abolished lactate-driven migration and FAO activity. In vivo, lactylation-deficient CPT1A mutants (K180R/K285R) attenuated lung metastasis and subcutaneous tumor growth in nude mice. This study reveals that lactate-CPT1A axis synergistically amplifies FAO to fuel PTC progression, suggesting CPT1A lactylation as a therapeutic vulnerability for metabolic intervention.

## Introduction

According to the 2022 global cancer statistics released by the International Agency for Research on Cancer (IARC) under the World Health Organization (WHO), thyroid cancer has risen to become the seventh most common malignant tumor worldwide (GLOBOCAN 2022) [1]. Notably, this disease exhibits significant gender disparity, with approximately 75% of cases occurring in females [2]. Among all thyroid cancer sub-types, papillary thyroid carcinoma (PTC) demonstrates the highest incidence, accounting for 80-90% of all thyroid malignancies [3]. Currently, over-treatment of indolent PTC has led to a dramatic surge in healthcare resource consumption [4]. Recent international consensus advocates for risk-stratified management, proposing active surveillance as an alternative to immediate surgery for low-risk PTC [5]. With advancements in molecular pathology, biomarkers such as *BRAF* and *TERT* promoter mutations have been incorporated into the AJCC staging system to guide prognostic evaluation and targeted therapy [6]. Nevertheless, existing biomarkers still suffer from insufficient predictive efficacy, highlighting an urgent need to identify novel molecular targets for precision diagnosis and treatment. Although PTC generally exhibits favorable prognosis, aggressive sub-types harboring molecular features such as concurrent *BRAF* and *TERT* promoter mutations or *TP53* mutations are prone to recurrence, distant metastasis, and even mortality [7]. PTC is characterized by multi-focality and early lymph node metastasis, with postoperative recurrence rates reaching 35% in patients exhibiting these features [8]. Therefore, elucidating the intrinsic mechanisms underlying PTC invasion and metastasis remains imperative.

Lactate, a glycolytic metabolite produced under anaerobic conditions, has long been dismissed as a metabolic waste product. However, in the 1920s, Otto Warburg observed that cancer cells avidly uptake glucose and preferentially convert it to lactate via glycolysis even in oxygen-rich environments, a phenomenon later termed the “Warburg effect“ [9]. Emerging evidence now reveals that lactate not only serves as a metabolic fuel transported into cells but also functions as a critical signaling molecule regulating diverse physiological and pathological processes [10]. Compared to normal tissues, tumor microenvironments exhibit markedly elevated lactate concentrations [11]. Tumor cells extensively import extracellular lactate, which is subsequently oxidized in the cytoplasm to generate NADH via the malate-aspartate shuttle, fueling mitochondrial oxidative phosphorylation and thereby driving cancer cell proliferation [12]. Intra-cellular lactate accumulation in cancer cells suppresses mitochondrial reactive oxygen species (ROS) production, conferring resistance to programmed cell death [13]. Moreover, lactate induces epithelial-mesenchymal transition (EMT), enhances migratory capacity, stimulates angiogenesis, facilitates immune evasion, and promotes metastatic dissemination [14–16]. Recent studies further implicate lactate in the development of therapeutic resistance across multiple cancer types [17, 18]. In addition, Professor Yingming Zhao’s team at the University of Chicago reported in *Nature* that lactate, beyond its role as a metabolic byproduct, can post-translationally modify lysine residues on histones, termed “lactylation“ [19]. Subsequent studies have revealed that lactylation is a widespread post-translational modification across diverse proteins. Functioning as an epigenetic regulator, lactylation participates in multiple physiological and pathological processes, including cancer progression, inflammatory and immune-related disorders, insulin resistance, and non-alcoholic fatty liver disease [20–22]. However, the precise mechanisms by which non-histone lactylation influences oncogenic phenotypes and its crosstalk with other signaling pathways remain poorly characterized, necessitating systematic investigations to unravel its biological significance in cancer biology.

Metabolic reprogramming represents a hallmark of tumor cells [23]. Under hypoxic conditions, tumor cells can uptake lactate from the microenvironment to drive lipid metabolic reprogramming. Studies demonstrate that lactate secreted by tumor-associated fibroblasts is absorbed by prostate cancer cells, where it enhances mitochondrial activity and fatty acid oxidation (FAO) to promote tumor progression [24]. FAO predominantly occurs in mitochondria, with carnitine palmitoyltransferase 1 (CPT1), including three isoforms (CPT1A, CPT1B, and CPT1C) [25]. Among these, CPT1A exhibits the highest affinity for carnitine and is ubiquitously expressed across human tissues [25]. Mounting evidence establishes CPT1A as a critical driver of tumor progression and a promising therapeutic target [26]. For instance, Xiong et al. demonstrated that CPT1A knockdown suppresses breast cancer cell invasion and lymphangiogenesis [27]. Schlaepfer et al. reported that etomoxir, an irreversible CPT1A inhibitor, significantly reduces prostate cancer cell viability [28]. However, whether lactate directly modulates CPT1A expression and the mechanistic basis of its pro-metastatic effects in PTC remain unexplored.

This study aims to elucidate the functional role of lactate accumulation in PTC progression and to decipher the underlying mechanism. Our data showed that lactate potentiates metastatic dissemination through CPT1A-mediated FAO. Further investigation revealed that lactate upregulates CPT1A though histone H3K18 lactylation (H3K18la)-dependent transactivation and CPT1A (at K180/K285) lactylation-dependent stabilization. These findings illustrate a mechanistic foundation for targeting the lactate-CPT1A axis to develop metabolic therapeutic strategies against PTC progression and metastasis.

## Results

### Elevated lactate levels promote PTC migration

**Fig. 1:**
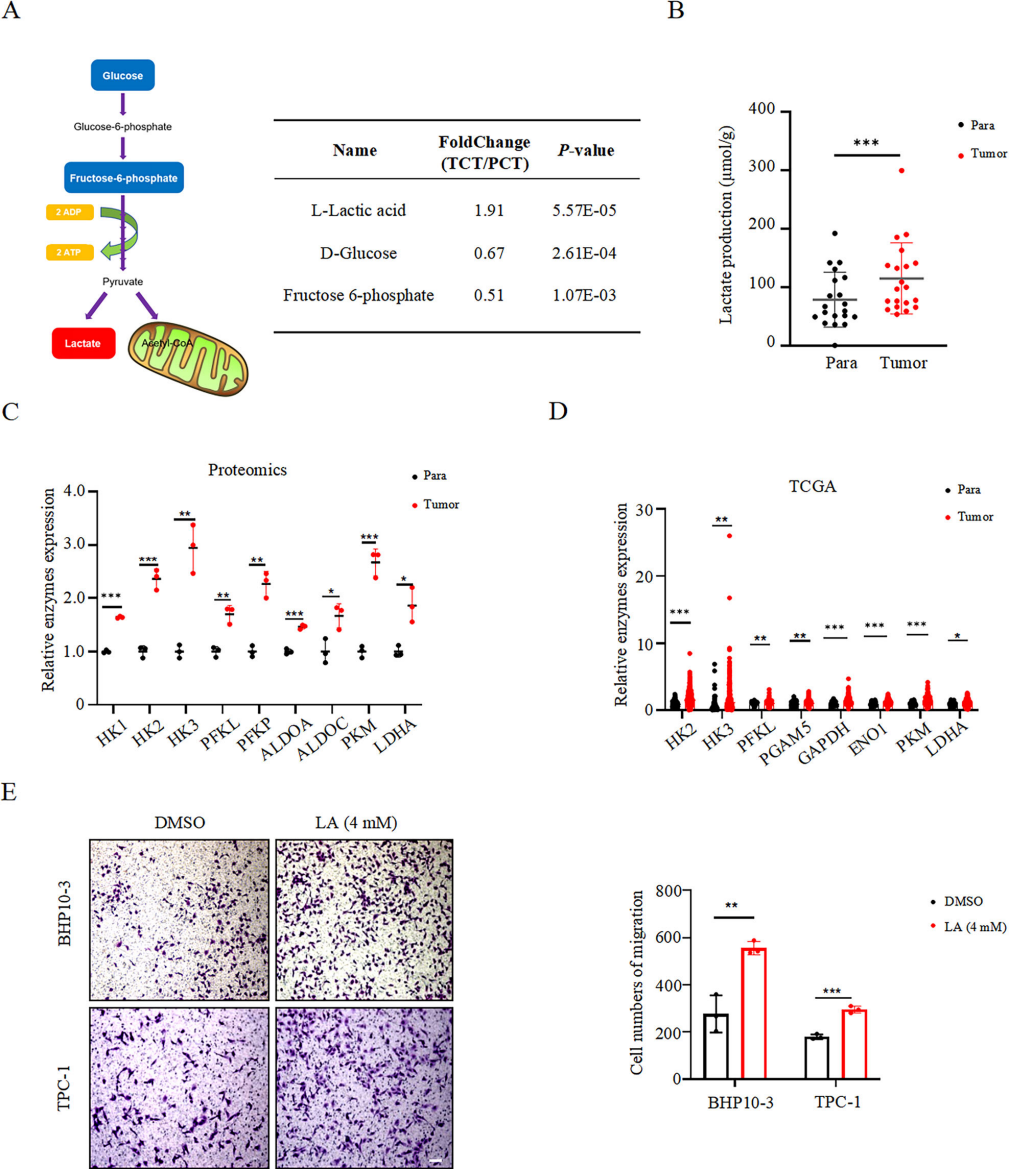
Elevated lactate levels promote tumor migration in PTC. **A** Metabolomic sequencing identified differentially expressed metabolites in PTC tissues. *Left panel*: schematic diagram of the glycolytic pathway; *right panel*: statistical plot of key differentially metabolites (*n* = 27). **B** Lactate content in PTC tissues and adjacent normal tissues was measured using a lactate assay kit. Data are presented as scatter plots, with each dot representing an individual patient sample (*n* = 20). **C** Proteomic profiling revealed expression levels of key glycolytic enzymes in PTC tissues. For proteomic sequencing, the 27 samples were pooled into 3 groups for analysis, hence each dot represents one pooled group (*n* = 3 technical replicates, each from 9 pooled patient samples). **D** TCGA database analysis of differentially expressed glycolysis-related genes in PTC. **E** Transwell migration assay of PTC cells treated with lactate (4 mM). *Left panel*: representative images (Scale bar: 200 μm); *right panel*: quantification of migrated cells (*n* = 3). All the data are presented as mean ± SD; *p < 0.05, **p < 0.01, and ***p < 0.001.

To systematically delineate the metabolic profile of PTC, we employed proteomic and metabolomic approaches to analyze protein and metabolite levels in 27 paired PTC tissues and adjacent non-tumor tissues [29]. Initial metabolomic profiling revealed a marked elevation of lactate levels in PTC tissues compared to matched adjacent non-tumor tissues (Fig. 1A). Subsequently, this result was validated using an independent cohort of 20 clinical specimens. As shown in Fig. 1B, lactate concentrations in each individual sample are presented as a scatter plot, clearly demonstrating the elevated levels in PTC tissues compared to adjacent normal tissues. Furthermore, proteomic analyses identified increased expression of key glycolytic enzymes in PTC tissues relative to adjacent normal tissues (Fig. 1C). For the proteomic data, where 27 samples were pooled into three groups for sequencing, each dot in Fig. 1C represents one pooled group, illustrating the consistent upregulation of glycolytic enzymes in PTC. These findings were further corroborated through bioinformatic interrogation of The Cancer Genome Atlas (TCGA) database, which revealed significant upregulation of core glycolytic pathway genes in PTC specimens compared to normal thyroid tissues (Fig. 1D). The concordant multi-omics evidence strongly implicates enhanced glycolytic flux and consequent lactate accumulation as characteristic metabolic features of PTC pathogenesis.

To investigate the effect of elevated lactate on the metastatic behavior of PTC cells, we selected two PTC cell lines, BHP10-3 and TPC-1, to perform transwell assays. These cells were treated with 4 mM lactate (the optimal concentration determined by dose-response experiments, see Methods) in culture medium and followed by seeded in the up-chamber for 24 h. Results demonstrated a significant increase in the number of migrating PTC cells in lactate-treated groups compared to the control group (Fig. 1E). These findings indicate that exogenous lactate markedly enhances the migratory ability of PTC cells.

### Lactate enhances fatty acid β-oxidation in PTC cells

**Fig. 2:**
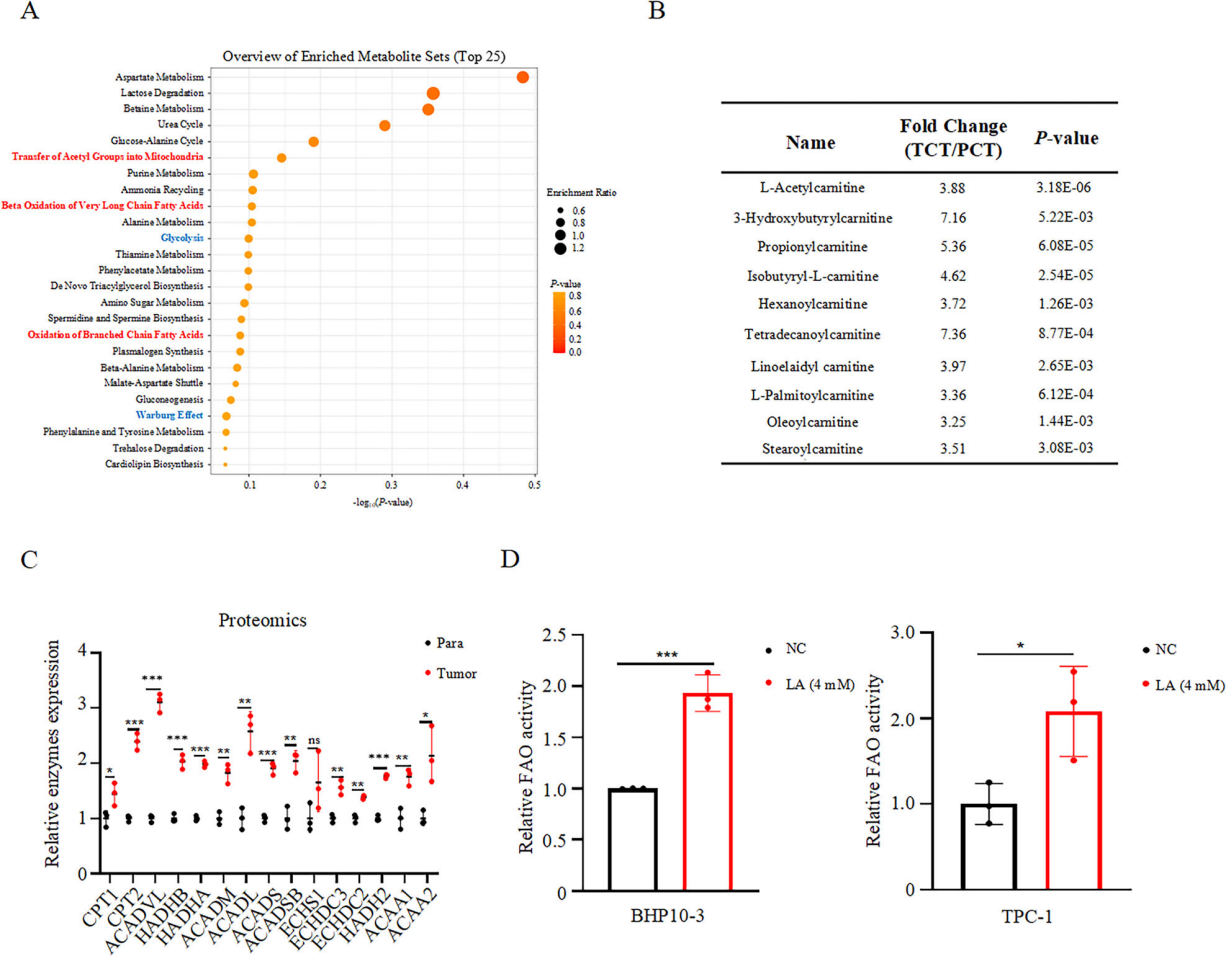
Lactate enhances fatty acid β-oxidation in PTC cells. **A** Metabolite set enrichment analysis of PTC tissues versus adjacent normal tissues. **B** Sequencing profiling of lipid metabolites showed increased carnitines in PTC tissues and adjacent normal tissues (*n* = 27). **C** Proteomic profiling revealed expression levels of key enzymes in the fatty acid β-oxidation pathway in PTC tissues versus adjacent normal tissues (*n* = 27). **D** Fatty acid β-oxidation activity was measured in BHP10-3 and TPC-1 cells treated with exogenous lactate (4 mM) using a β-oxidation assay kit (*n* = 3). All the data are presented as mean ± SD; ns, not significant; * *p* < 0.05, **p < 0.01, and ***p < 0.001.

Given the established interplay between the Warburg effect and lipid metabolic reprogramming, we further investigated the lactate-regulated metabolic mechanisms underlying PTC progression [9]. Untargeted metabolomic enrichment analysis of 27 paired PTC and adjacent non-tumor tissues revealed striking enrichment of fatty acid oxidation related pathways, including β-oxidation of very long chain fatty acids, oxidation of branched chain fatty acids and transfer of acetyl groups into mitochondria (Fig. 2A). Subsequent lipid metabolomic profiling confirmed significant accumulation of multiple long-chain acylcarnitines in PTC specimens (Fig. 2B). Further, proteomic analyses demonstrated pronounced upregulation of key enzymes involved in fatty acid β-oxidation (FAO), especially CPT1, the rate limiting enzyme of FAO (Fig. 2C). Collectively, these multi-omics findings indicate enhanced FAO capacity in PTC, a metabolic feature potentially linked to lactate-mediated tumor progression. To validate lactate’s regulatory role in FAO, FAO activity assays were performed in BHP10-3 and TPC-1 cells treated with 4 mM exogenous lactate. The results revealed significantly increased FAO activity in lactate-treated cells compared to untreated controls (Fig. 2D). These results demonstrate that exogenous lactate enhances FAO capacity in PTC cells.

### Lactate promotes PTC cell migration via enhanced fatty acid β-oxidation

**Fig. 3:**
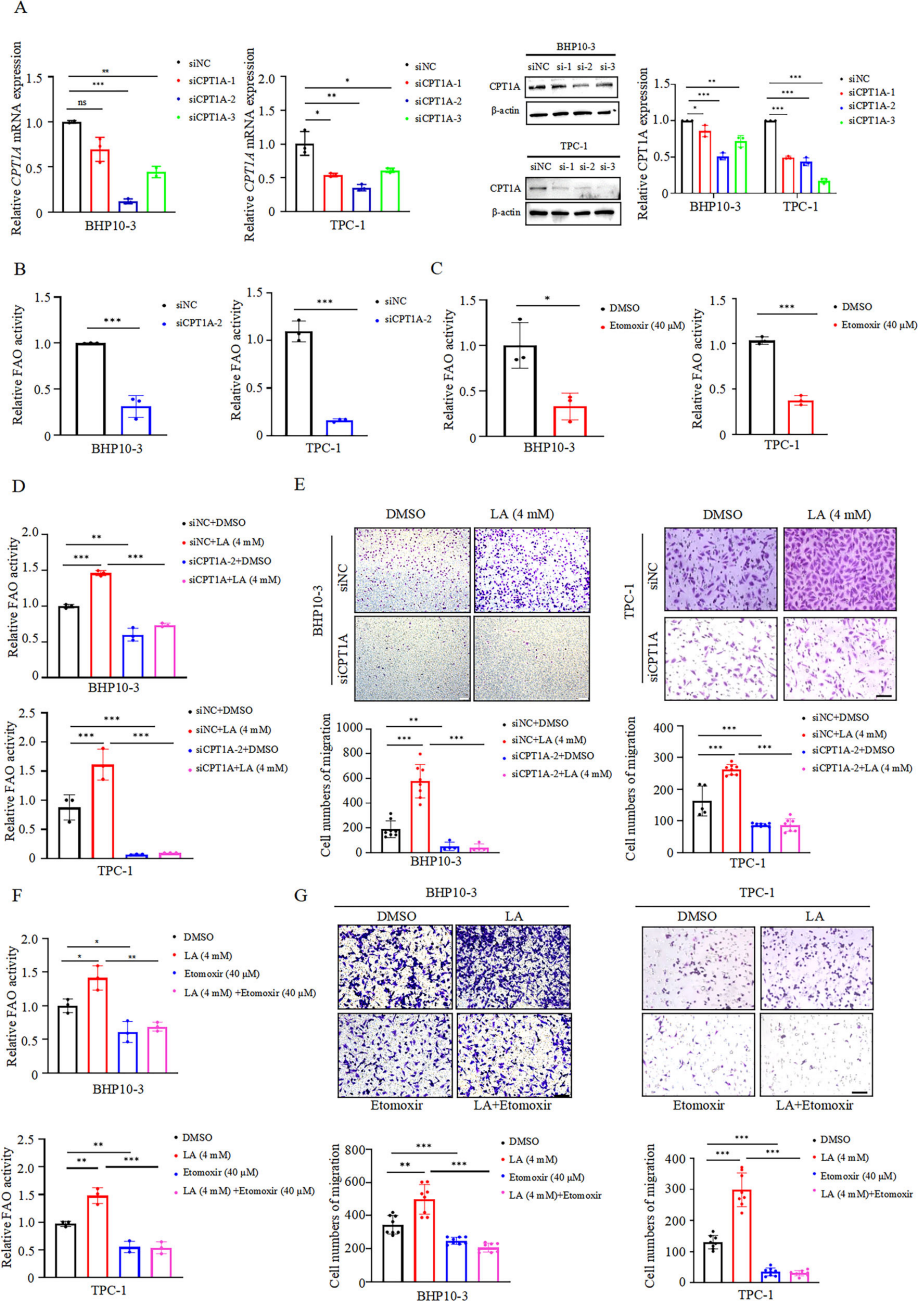
Lactate promotes PTC cell migration *via* enhanced fatty acid β-oxidation. **A** Knockdown efficiency of siRNAs-targeting CPT1A was validated by RT-qPCR and Western blotting. *Left panel*: RT-qPCR analysis (*n* = 3); *right panel*: representative Western blot images and quantitative data. **B** Fatty acid β-oxidation activity in CPT1A-knockdown BHP10-3 cells and TPC-1 cells was measured using a β-oxidation assay kit (*n* = 3). **C** Fatty acid β-oxidation activity in BHP10-3 cells and TPC-1 cells treated with Etomoxir (40 μM) was assessed by β-oxidation assay (*n* = 3). Fatty acid β-oxidation activity (**D**) and transwell migration assay (**E**) in CPT1A-knockdown BHP10-3 cells and TPC-1 cells treated with lactate (4 mM) (*n* = 3). E *Upper panel*: representative images (Scale bar: 100 μm); E *lower panel*: quantification of migrated cells (*n* = 8). Fatty acid β-oxidation activity (**F**) and transwell migration assay (**G**) of BHP10-3 cells and TPC-1 cells treated with Etomoxir (40 μM) and lactate (4 mM). G *Upper panel*: representative images (Scale bar: 100 μm); G *lower panel*: quantification of migrated cells (*n* = 8). All the data are presented as mean ± SD; ns, not significant; * *p* < 0.05, **p < 0.01, and ***p < 0.001.

To delineate the functional role of FAO in lactate-driven PTC cell migration, we employed genetic silencing and pharmacological inhibition strategies. Specific siRNAs targeting CPT1A were designed and validated via RT-qPCR and Western blot. Among them siCPT1A-2 showed more obvious knockdown efficiency than the other two siRNAs (Fig. 3A). In BHP10-3 cells and TPC-1 cells, knockdown of CPT1A or treatment with CPT1A inhibitor etomoxir significantly attenuated fatty acid β-oxidation activity compared to controls (Fig. 3B, C). Importantly, FAO assays confirmed that CPT1A silencing reversed lactate-mediated augmentation of fatty acid catabolism (Fig. 3D). Subsequently, transwell assays revealed that knockdown of CPT1A abrogated lactate-induced migratory enhancement in lactate-treated group (Fig. 3E). Similarly, etomoxir treatment demonstrated that inhibition of FAO reversed lactate-driven migration (Fig. 3F, G). These data collectively indicate that fatty acid β-oxidation as a critical mediator of lactate-induced PTC cell migration.

### Lactate upregulates CPT1A to enhance fatty acid β-oxidation in PTC cells

**Fig. 4:**
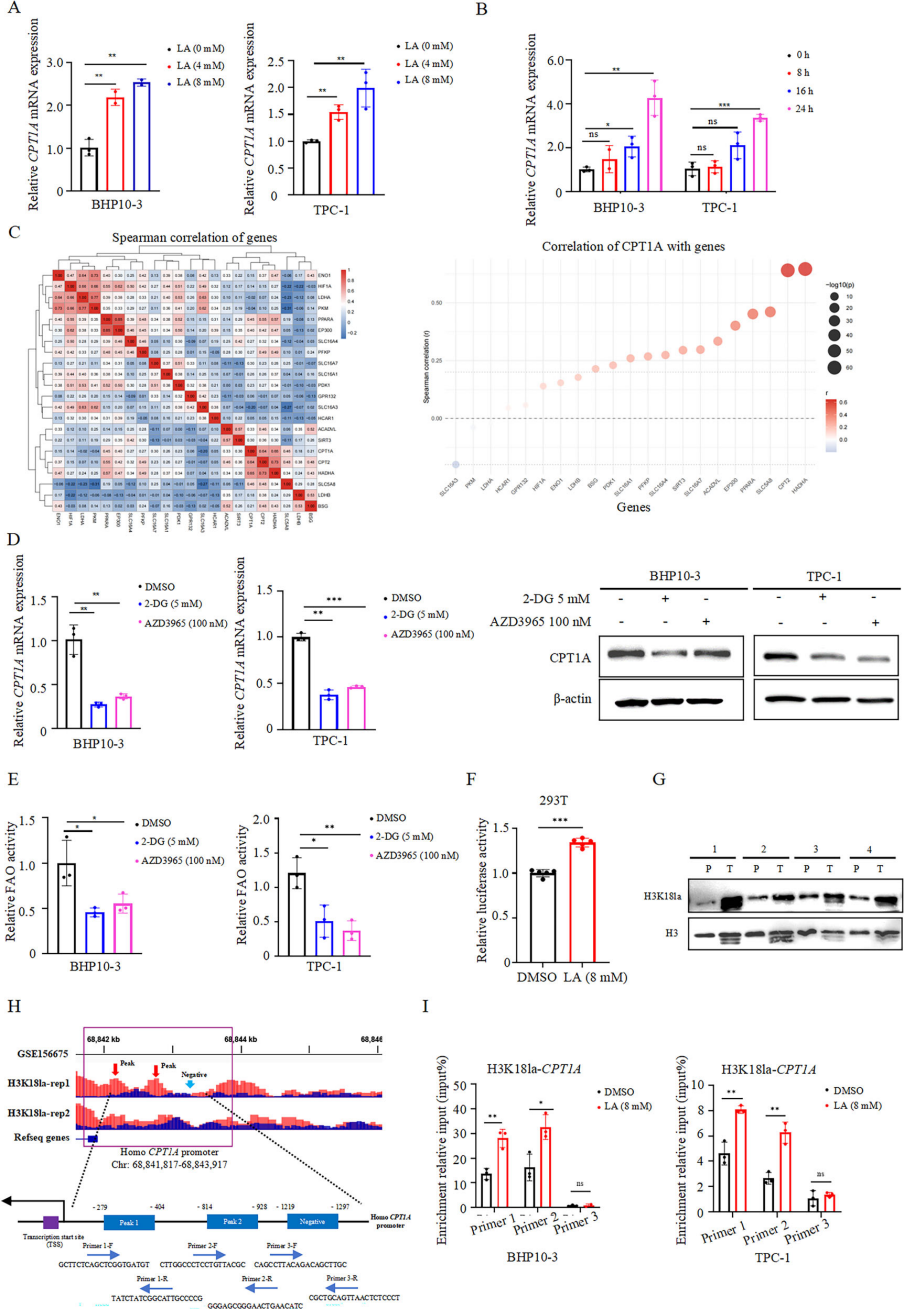
Lactate upregulates CPT1A via H3K18la. **A** *CPT1A* mRNA levels in BHP10-3 and TPC-1 cells treated with lactate at different concentrations (4 mM and 8 mM) were analyzed by RT-qPCR (*n* = 3). **B** Time-dependent changes in *CPT1A* mRNA levels were measured by RT-qPCR in BHP10-3 and TPC-1 cells treated with lactate (4 mM) for 0, 8, 16, or 24 h (*n* = 3). **C** Correlation analysis of *CPT1A* with key metabolic genes in PTC based on TCGA THCA transcriptomic data. *Left panel*: Heatmap showing the expression correlation matrix between *CPT1A* and selected genes involved in fatty acid oxidation (FAO), lactate transport, glycolysis, and metabolic regulation. Red indicates positive correlation, blue indicates negative correlation, with color intensity proportional to the Pearson correlation coefficient (r). *Right panel*: Bubble plot summarizing the correlation of *CPT1A* with individual genes. The x-axis represents the Pearson correlation coefficient (r), the y-axis lists the genes, and the bubble size is proportional to the statistical significance (-log10(*p*-value)). Bubbles are colored red for positive correlations and blue for negative correlations. Data were analyzed using RStudio. **D** *CPT1A* mRNA and protein levels in BHP10-3 cells and TPC-1 cells treated with 2-DG (5 mM) or AZD3965 (100 nM) were detected by RT-qPCR (*n* = 3). *Left panel*: RT-qPCR analysis (*n* = 3); *right panel*: representative Western blot images. **E** Fatty acid β-oxidation activity in BHP10-3 cells and TPC-1 cells treated with 2-DG (5 mM) or AZD3965 (100 nM) was assessed using the β-oxidation assay kit (*n* = 3). **F** Dual-luciferase reporter assays were used to evaluate CPT1A promoter activity in 293 T cells treated with lactate (8 mM) (*n* = 5). **G** Western blot analysis was performed to compare H3K18la expression levels between PTC tissues and adjacent normal tissues. **H** Representative UCSC Genome Browser view of H3K18la ChIP-seq signals (GSE156675) at the CPT1A genomic locus. The transcription start site (TSS) and direction of transcription are indicated. Two distinct H3K18la enrichment peaks within the CPT1A promoter region are highlighted (Peak 1 and Peak 2). A region approximately 1.2 kb upstream of the TSS showing no H3K18la enrichment was selected as a negative control (Negative). The positions of the qPCR amplicons used in (**H**) are indicated below the peaks. **I** ChIP-qPCR validation of H3K18la enrichment at the CPT1A promoter in BHP10-3 and TPC-1 cells treated with or without lactate (8 mM) for 24 h. Enrichment was measured using primers targeting Peak 1, Peak 2, and the Negative control region. All the data are presented as mean ± SD; ns, not significant; * *p* < 0.05, **p < 0.01, and ***p < 0.001.

To elucidate the molecular mechanism underlying lactate-driven fatty acid β-oxidation, we investigated the effects of exogenous lactate on CPT1A expression. RT-qPCR analysis demonstrated that lactate treatment (0, 4, 8 mM for 24 h) induced dose-dependent upregulation of *CPT1A* mRNA levels in PTC cells (Fig. 4A). Notably, 8 mM lactate was used here to clearly illustrate the dose-dependent trend, while 4 mM was the standard concentration for functional assays to avoid potential cytotoxic effects (Supplementary Fig. 1). Meanwhile time-course experiments (4 mM lactate for 24 h) revealed progressive time-dependent elevation of *CPT1A* mRNA levels (Fig. 4B). These results indicate that exogenous lactate enhances CPT1A transcription. To further explore the transcriptional relationship between *CPT1A* and the lactate metabolism network in PTC, we performed an extended correlation analysis using TCGA THCA transcriptomic data. As shown in Fig. 4C (left panel), a heatmap of selected genes involved in FAO, lactate transport, glycolysis, and metabolic regulation revealed a broad co-expression pattern with *CPT1A*. Specifically, *CPT1A* expression was strongly and positively correlated with key FAO enzymes, including *HADHA* (r = 0.65, *p* = 5.3e–61), *CPT2* (r = 0.64, *p* = 1.3e–59), and *ACADVL* (r = 0.33, *p* = 1.3e–14). Notably, *CPT1A* also exhibited significant positive correlations with lactate uptake transporters, such as *SLC5A8* (*SMCT1*) (r = 0.46, *p* = 6.7e–28), *SLC16A7* (*MCT2*) (r = 0.30, *p* = 9.1e–12), and *SLC16A1* (*MCT1*) (r = 0.26, *p* = 3.1e–9), as well as with the MCT chaperone *BSG* (*CD147*) (r = 0.21, *p* = 1.2e–6). In contrast, the lactate efflux transporter *SLC16A3* (*MCT4*) was significantly negatively correlated with *CPT1A* (r = –0.20, *p* = 6.2e–6). Furthermore, *CPT1A* positively correlated with transcriptional regulators of FAO and lactylation, including *PPARA* (r = 0.45, *p* = 9.7e–27) and the lactyltransferase *EP300* (r = 0.40, *p* = 6.1e–21). The bubble plot in Fig. 4C (right panel) summarizes these correlation coefficients and their statistical significance, highlighting the robust association of *CPT1A* with genes involved in FAO and lactate influx. These transcriptomic observations suggest that *CPT1A* expression is tightly coordinated with the lactate metabolic network in PTC, providing a clinical context for our subsequent mechanistic investigations. To functionally validate the regulatory role of lactate on *CPT1A* expression, we treated PTC cells with pharmacological inhibitors targeting lactate production (2-deoxy-D-glucose, 2-DG, 5 mM) and transportation (AZD3965, 100 nM). Both inhibitors markedly reduced *CPT1A* mRNA expression in BHP10-3 and TPC-1 cells (Fig. 4D), and correspondingly decreased FAO activity (Fig. 4E). Collectively, these findings suggest that lactate can regulate CPT1A expression to modulate FAO in the PTC cell lines tested.

Emerging evidence highlights lactate’s role in epigenetic regulation via histone lactylation, particularly at histone H3 lysine 18 (H3K18la), a modification linked to chromatin relaxation and transcriptional activation [30]. Therefore, our study focused on this specific lactylation mark to directly link lactate metabolism to epigenetic regulation of CPT1A. To investigate whether lactate regulates CPT1A expression through this mechanism, we first cloned the CPT1A promoter sequence into a dual-luciferase reporter plasmid. Co-transfection of this construct with a Renilla luciferase control plasmid into 293 T cells, followed by lactate treatment (8 mM, 24 h, a concentration determined to be optimal for this cell line, see Methods), revealed significantly enhanced CPT1A promoter activity (Fig. 4F). Western blot analysis of histone extracts from four paired PTC and adjacent tissues demonstrated elevated H3K18la levels in tumors (Fig. 4G). Interrogation of publicly available H3K18la ChIP-seq data (GSE156675) via the UCSC Genome Browser revealed pronounced enrichment of H3K18la at the CPT1A promoter region. As shown in Fig. 4H, two distinct H3K18la enrichment peaks were identified within the CPT1A promoter (designated as Peak 1 and Peak 2), while a region approximately 1.2 kb upstream of the transcription start site showed no enrichment and was selected as a negative control region (Negative). To experimentally validate these findings, we performed ChIP-qPCR assays in both BHP10-3 and TPC-1 cells using primers specifically designed to target Peak 1, Peak 2, and the Negative region. Lactate treatment (8 mM) significantly increased H3K18la occupancy at both Peak 1 and Peak 2 in both cell lines, whereas no enrichment was detected at the Negative region under any condition (Fig. 4I). These results mechanistically link lactate-induced histone lactylation to site-specific transcriptional activation of CPT1A.

### Lactate inhibits CPT1A degradation by enhancing its lactylation

**Fig. 5:**
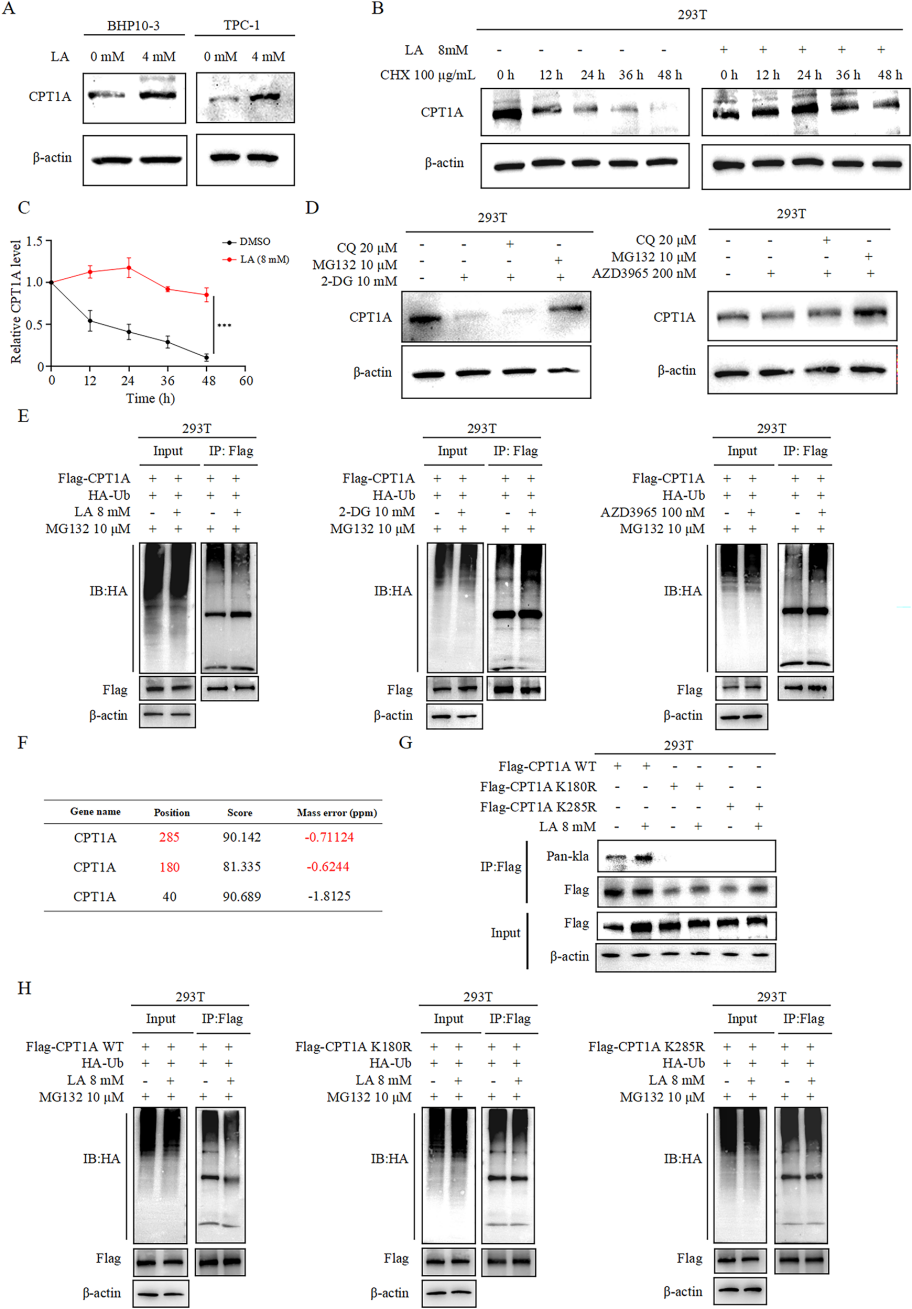
Lactate inhibits CPT1A degradation by enhancing its lactylation. **A** CPT1A protein levels in BHP10-3 cells and TPC-1 cells treated with lactate at different concentrations (4 mM) was determined by Western blot. **B** Western blot analysis of CPT1A protein levels in 293 T cells treated with or without lactate (8 mM) and cycloheximide (CHX, 100 µg/mL) for 0, 12, 24, 36, or 48 h. **C** Quantification of CPT1A degradation kinetics over time according (**B**). **D** Western blot detection of CPT1A protein in 293 T cells treated with 2-DG (10 mM) or AZD3965 (200 nM) for 24 h, followed by MG132 (10 µM) or chloroquine (CQ, 20 µM) treatment. **E** Immunoprecipitation were performed to assess ubiquitination levels of CPT1A in 293 T cells overexpressing CPT1A and treated with lactate (8 mM), 2-DG (10 mM), or AZD3965 (100 nM). **F** Two lactylation sites (K180 and K285) on CPT1A were selected for mutagenesis based on LC-MS/MS data from hepatocellular carcinoma studies (mass error < 1 ppm). **G** Lactylation levels of CPT1A were evaluated by immunoprecipitation in 293 T cells overexpressing WT or mutant CPT1A (K180R/K285R) and treated with lactate (8 mM). **H** Ubiquitination levels of CPT1A were measured by immunoprecipitation in 293 T cells overexpressing WT or mutant CPT1A (K180R/K285R) and treated with lactate (8 mM). All the data are presented as mean ± SD; ns, ***p < 0.001.

While the aforementioned findings demonstrate that lactate enhances CPT1A expression by promoting its promoter activity, protein abundance is also regulated by degradation pathways. To investigate whether lactate affects CPT1A protein stability, we first treated BHP10-3 cells and TPC-1 cells with 4 mM lactate and observed a elevation in CPT1A protein levels via Western blot (Fig. 5A). Cycloheximide (CHX) chase assays revealed that lactate treatment (8 mM, a concentration determined to be optimal for this cell line, see Methods) significantly delayed CPT1A protein degradation over 48 h period in 293 T cells (Fig. 5B, C), indicating lactate extends CPT1A protein half-life. To delineate the degradation pathway involved, cells were pretreated with lactate synthesis inhibitor 2-DG (10 mM) or lactate transporter inhibitor AZD3965 (200 nM), followed by co-treatment with the proteasome inhibitor MG132 (10 μM) or lysosome inhibitor chloroquine (CQ, 20 μM). Western blot analysis demonstrated that 2-DG and AZD3965 accelerated CPT1A degradation, which was rescued by MG132 but not CQ (Fig. 5D), suggesting lactated-regulated CPT1A stability is dependent on the ubiquitin-proteasome system. Co-IP assays in CPT1A- and ubiquitin (Ub)-overexpressing cells further confirmed that lactate treatment reduced CPT1A ubiquitination, whereas 2-DG and AZD3965 increased ubiquitin conjugation (Fig. 5E). These results demonstrated that lactate stabilizes CPT1A protein by suppressing ubiquitin-proteasome-mediated degradation, thereby prolonging its half-life and amplifying its metabolic functions in PTC cells.

To delineate the molecular mechanism underlying lactate-mediated stabilization of CPT1A protein, we focused on protein lactylation modifications. Previous studies have demonstrated that lactate induces lactylation of specific amino acid residues in cellular proteins, thereby suppressing their degradation and enhancing protein abundance [31]. Leveraging published hepatocellular carcinoma LC-MS/MS data [32]. we predicted two putative lactylation sites on CPT1A: lysine 180 (K180) and lysine 285 (K285) (Fig. 5F). Based on the sequence information of the lactylation modification sites, point mutation plasmids were constructed. Overexpression of wild-type (WT) or mutant CPT1A in 293 T cells, followed by lactate treatment (8 mM), revealed that lactate treatment enhanced lactylation levels in WT CPT1A, whereas no lactylation was detected in either mutant (Fig. 5G), confirming K180 and K285 as bona fide lactylation sites. To assess the functional impact of lactylation on protein stability, 293 T cells co-expressing HA-tagged ubiquitin (HA-Ub) with either WT or mutant CPT1A were treated with lactate (8 mM). Co-IP analysis demonstrated that lactate significantly reduced ubiquitination of WT CPT1A but had no effect on the ubiquitination of K180R or K285R mutants (Fig. 5H). These results suggest that lactylation at K180 and K285 of CPT1A suppresses its ubiquitin-proteasomal degradation, thereby stabilizing CPT1A to extend its functional lifespan.

### Inhibiting CPT1A lactylation attenuates its pro-tumor effects in PTC

**Fig. 6:**
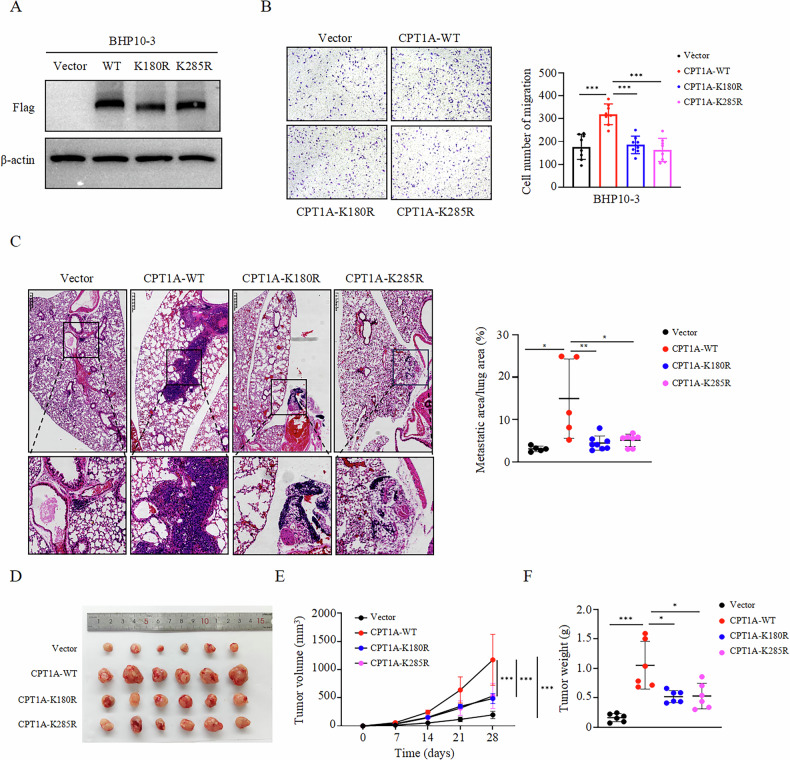
Inhibiting CPT1A lactylation attenuates its pro-tumor effects in PTC. **A** Stable BHP10-3 cell lines overexpressing WT or mutant CPT1A (K180R/K285R) were established and Western blot analysis confirmed CPT1A expression. **B** Transwell assay of BHP10-3 cells stably overexpressing WT or mutant CPT1A. *Left panel*: representative images (Scale bar: 100 μm); *right panel*: quantification of migrated cells (*n* = 8). **C** Lung metastasis model was established by tail vein injection of control (*n* = 5), WT- (*n* = 5), or mutant CPT1A (K180R/K285R)- (*n* = 8 per group) expressing BHP10-3 cells in mice. *Left panel*: H&E-stained lung sections showing metastatic nodules (Scale bar: 400 μm). *Right panel*: quantitative analysis of metastatic area (% of total lung area). **D**-**F** Subcutaneous xenograft tumor model was generated by injecting control, WT-, or mutant CPT1A-expressing BHP10-3 cells into nude mice (*n* = 6). Tumor growth was monitored until day 28, followed by excision for gross imaging (**D**), growth curves (**E**), and weight quantification (**F**). All the data are presented as mean ± SD; * *p* < 0.05, **p < 0.01, and ***p < 0.001. Supplementary Fig. 1. Morphological changes of PTC cells treated with increasing concentrations of lactate. BHP10-3 cells (**A**) and TPC-1 cells (**B**) were treated with sodium L-lactate at the indicated concentrations (0, 4, 8, and 12 mM) for 24 hours. Cell morphology was observed and photographed under a light microscope. Concentrations of 8 mM and above induced noticeable cellular stress and morphological changes, including cell shrinkage and reduced adherence, whereas 4 mM lactate maintained normal cell morphology comparable to the untreated control (Scale bar: 100 μm).

To validate the functional role of CPT1A lactylation in PTC progression, we performed experiments to assess its impact on tumor cell metastasis. Lentiviral vectors overexpressing WT or lactylation site-mutated CPT1A (K180R/K285R) were constructed. Stable PTC cell lines expressing WT or mutant CPT1A were established via lentiviral infection, with Western blot confirming protein expression (Fig. 6A). Transwell migration assays demonstrated that mutating lactylation sites significantly attenuated CPT1A-driven cell migration (Fig. 6B). For in vivo validation, a lung metastasis model in nude mice were established. The results revealed that WT CPT1A overexpression markedly increased metastatic lesion area compared to the vector control, whereas K180R/K285R mutants exhibited reduced metastatic burden (Fig. 6C). Additionally, subcutaneous xenograft models showed that WT CPT1A promoted tumor growth, while lactylation-deficient mutants (K180R/K285R) abrogated this tumor promoting effect (Fig. 6D-F). These findings collectively demonstrate that CPT1A lactylation drives PTC metastasis and growth in vivo.

## Discussion

With rising incidence, approximately 10–15% of aggressive PTC cases develop recurrence and distant metastasis, which can be fatal. However, the molecular mechanisms underlying their metastatic propensity remain poorly characterized [7]. Basing on our previous multi-omics analysis of 27 paired PTC tissues revealing profound lactate metabolic dysregulation and β-oxidation activation, we demonstrate that lactate drives PTC metastasis via lactylation-mediated regulation of CPT1A. On one hand, lactate promotes CPT1A transcriptional activation by enhancing histone H3K18 occupancy at the CPT1A promoter. On the other hand, lactate induces K180/K285 lactylation on CPT1A protein, which stabilizes CPT1A by suppressing ubiquitin-proteasomal degradation. These coordinated epigenetic and post-translational modifications synergistically amplify CPT1A-driven FAO to fuel metastatic progression of PTC. Our findings not only decipher a lactate-CPT1A regulatory axis central to PTC metabolic reprogramming but also identify CPT1A lactylation as a novel diagnostic biomarker and therapeutic vulnerability. Targeting CPT1A lactylation with specific inhibitors may represent a precision strategy to combat aggressive PTC.

The discovery of the Warburg effect has revolutionized our understanding of lactate as a central regulator in cellular metabolism and signaling. Beyond serving as a metabolic substrate, lactate functions as a pleiotropic signaling molecule with ubiquitous roles in physiological and pathological processes [10]. Cellular lactate transport is primarily mediated by monocarboxylate transporters (MCTs)—MCT1, MCT2, MCT3, and MCT4 [33, 34]. Elevated lactate levels, a hallmark of the tumor microenvironment [35]. This oncometabolite directly and indirectly regulates malignant progression across cancer types by modulating proliferation, metastasis, and chemoresistance [36–38]. For instance, lactate activates the PI3K/Akt-cAMP-CREB axis to drive pro-angiogenic factor expression in breast cancer [36]. Our multi-omics analysis revealed enhanced glycolytic activation in PTC, characterized by upregulated metabolic enzymes and significantly elevated lactate levels compared to adjacent tissues. Functional validation demonstrated that exogenous lactate treatment markedly increased PTC cell migration in vitro. These findings collectively demonstrate that lactate accumulation as a critical metabolic driver of PTC progression, providing mechanistic insights into thyroid carcinogenesis through dysregulated tumor metabolism.

Emerging evidence highlights lactate’s capacity to reprogram tumor lipid metabolism, with recent studies demonstrating its role in enhancing FAO across malignancies [39]. Our integrated proteomic and metabolomic profiling of PTC tissues revealed pronounced Warburg effect amplification and significant enrichment of FAO-associated pathways in differentially expressed genes. Functional validation showed exogenous lactate administration markedly enhanced β-oxidation capacity in PTC cells. This observation aligns with Ippolito et al. discovery that lactate from cancer-associated fibroblasts fuels prostate cancer lipogenesis via citrate-mediated lipid droplet formation, which subsequently sustains mitochondrial oxidative phosphorylation under hypoxia [24]. To delineate lactate-FAO interplay in PTC pathogenesis, we employed CPT1A siRNAs and pharmacological inhibition, demonstrating that FAO blockage reverses lactate-driven migration enhancement. These results indicate that lactate-mediated FAO potentiation as a critical mechanism underlying PTC progression.

As the rate-limiting enzyme governing mitochondrial fatty acid import during β-oxidation, CPT1A plays indispensable roles in maintaining physiological homeostasis [40, 41]. In tumor, CPT1A-mediated lipid metabolic reprogramming enables cancer cell survival under nutrient-deprived or hypoxic stress [42]. Nandi et al. demonstrated CPT1A suppression enhances anti-ErbB2 immunotherapy in breast cancer by remodeling the immune microenvironment [43]. Zhu et al. revealed that CPT1A exerts non-canonical lysine succinyltransferase activity to catalyze succinylation of lysine 302 (K302) on mitochondrial fission factor, thereby suppressing its ubiquitination-mediated degradation and driving ovarian cancer progression through stabilization of mitochondrial fission machinery [44]. In this study, lactate treatments in PTC cells induced dose-responsive CPT1A upregulation at both transcriptional and protein levels. While, pharmacological blockage of lactate transport/production pathways suppressed CPT1A expression and attenuated FAO activity. These findings suggest that lactate as a novel upstream regulator of CPT1A in PTC, wherein lactate-induced CPT1A overexpression enhances β-oxidation capacity to fuel PTC progression.

Since the inaugural report by *Nature* in 2019 demonstrating lactate-induced histone lactylation, accumulating evidence has revealed widespread protein lactylation in diverse tumor cells, wherein lactate is enzymatically converted to lactoyl-CoA and transferred to substrate proteins via “writer” enzymes, including P300, GCN5, TIP60, and KATs [19, 20, 45, 46]. Recent studies identified alanyl-tRNA synthetase (AARS) as a novel lactylation “writer” that directly recognizes lactate to generate lactyl-adenylate for subsequent modifications [47, 48]. Notably, lactylation is classified into three isomeric forms (L-lactylation, D-lactylation, and N-ε-carboxyethylation) based on substrate specificity and further categorized as histone or non-histone lactylation, with histone lactylation critically regulating gene transcription [49]. Our study demonstrates that lactate positively regulates CPT1A transcription via H3K18la. H3K18la binds to the CPT1A promoter to promote its transcription. Beyond histones, non-histone lactylation plays a pivotal role in tumor progression by modulating protein-protein interactions, subcellular localization, enzymatic activity, and protein stability [50–54]. For instance, Zhang et al. demonstrated that lactate-induced lactylation of insulin-like growth factor 1 receptor (IGF1R) enhances its stability, facilitating IGF1 binding to promote lung cancer progression [55]. In this study, Co-IP assays confirmed lactylation of CPT1A, which was abolished by CPT1A mutation (K180R/K285R). CHX assays demonstrated that lactate significantly decelerated CPT1A degradation, while proteasomal inhibition with MG132 reversed lactate-depletion-induced CPT1A degradtion. Lactate markedly reduced CPT1A ubiquitination, an effect eliminated by CPT1A mutation. Collectively, these findings demonstrate that lactate stabilizes CPT1A by augmenting its lactylation to suppress ubiquitin-proteasomal degradation. Furthermore, in vitro and in vivo functional assays revealed that blocking CPT1A lactylation attenuated its pro-tumorigenic effects on PTC proliferation and migration. This study proposes a novel mechanism by which lactate-driven CPT1A lactylation may contribute to PTC progression, suggesting a potential therapeutic target for further investigation.

## Materials and methods

### Clinical samples

From March to June 2021, 53 tissue samples were consecutively collected from patients who underwent total thyroidectomy or unilateral lobectomy at Qilu Hospital, Shandong University, with preoperative FNA-confirmed PTC. All participants provided written informed consent. The cohort included 44 females and 9 males, with a mean age of 45.1 ± 8.5 years. Inclusion criteria were: (1) absence of hypertension, diabetes, coronary heart disease, allergic diseases, or metabolic disorders; (2) no history of thyroid surgery or hormone therapy; (3) intraoperative specimen dimensions meeting postoperative testing standards; and (4) histopathological confirmation of PTC. Tissue samples were immediately snap-frozen in liquid nitrogen and stored at –80 °C for subsequent analyses. Detailed patient characteristics are provided in our previous work [29]. This study was approved by the Ethics Committee of Qilu Hospital, Shandong University (KYLL-2021(ZM)-028).

### Cell lines

Human PTC cell lines BHP10-3 (provided by Prof. Zhiyan Liu, Shanghai Sixth People’s Hospital). TPC-1, as well as human embryonic kidney 293 T cells, were obtained from the Cell Bank of the Chinese Academy of Sciences. BHP10-3 and TPC-1 cells were cultured in RPMI-1640 medium (Gibco, C11875500BT) supplemented with 10% fetal bovine serum (FBS, Gibco, 10270-106), while 293 T cells were maintained in DMEM medium (Gibco, C11995500BT) containing 10% FBS. All cells were incubated at 37 °C in a humidified atmosphere with 5% CO₂. BHP10-3 and TPC-1 are well-characterized human PTC cell lines commonly used to study thyroid cancer progression. Based on our preliminary screening and public database analysis, both lines exhibit robust migratory capacity and express detectable levels of CPT1A, making them suitable models for investigating the lactate-CPT1A axis.

### Plasmids, primers, lentiviruses, and siRNAs

Plasmids pcDNA3.1-3×flag-CPT1A were purchased from Miaolingbio (Wuhan, China). Mutant plasmids (pcDNA3.1-3×flag-CPT1A-K180R/K285R) were obtained from Weizhen Biosciences (Shandong, China). Lentiviruses for stable overexpression (LV-CPT1A-WT, LV-CPT1A-K180R/K285R) and control vectors were procured from Weizhen Biosciences (Shandong, China). The siRNAs targeting CPT1A and scramble controls were synthesized by SynthBio (Shandong, China) and the sequences were shown in Supplementary Table 1. For transient and rapid knockdown to assess the acute functional effects of CPT1A loss, we employed specific siRNAs. This approach minimizes potential long-term compensatory metabolic adaptations that might occur in stable knockout models generated by shRNA or CRISPR/Cas9, allowing for a more direct assessment of CPT1A’s role in lactate-driven phenotypes.

### Lactate treatment and concentration selection

To determine the optimal lactate concentration for functional assays, BHP10-3 and TPC-1 cells were treated with increasing concentrations of sodium L-lactate (0, 4, 8, 12 mM) for 24 hours. Cell morphology and viability were monitored. Concentrations of 8 mM and above induced noticeable cellular stress and morphological changes (Supplementary Fig. 1). 4 mM lactate significantly enhanced these phenotypes without overt cytotoxicity. Therefore, 4 mM lactate was selected for subsequent experiments in PTC cell lines unless otherwise specified. For mechanistic studies in 293 T cells, which are more resilient, 8 mM lactate was used to achieve a robust and consistent effect, as determined by preliminary dose-response experiments.

### Lactate content measurement

Tissue lactate levels were quantified using a lactate assay kit (Solarbio, BC2235). Briefly, 0.1 g of PTC or adjacent tissues was homogenized in 1 mL extraction buffer I, followed by centrifugation at 12,000×g for 10 min at 4 °C. Supernatants were mixed with extraction buffer II and centrifuged again. Lactate concentrations were determined via enzymatic reactions at 570 nm using a microplate reader, with calculations based on a standard curve.

### Transwell assay

Cell migration was assessed using Transwell chambers (Corning, 354578). Cells (4 × 10⁴) in serum-free medium were seeded into the upper chamber, while the lower chamber contained 10% FBS medium. After 24 h, migrated cells were fixed with methanol, stained with crystal violet, and captured with a microscope. The migrated cell numbers were countered using ImageJ software.

### FAO activity measurement

FAO activity was measured using a commercial kit (AssayGenie, BR00001). Cells (1 × 10^6^) were lysed, and supernatants were incubated with FAO substrate at 37 °C for 90 min. Absorbance at 492 nm was measured, and enzyme activity was normalized to protein concentration.

### Plasmid transfection and RNA interference

Cells were transfected with plasmids or siRNA using Lipo8000 (Beyotime, C0533) in Opti-MEM medium (Gibco, 31985070). Transfected cells were harvested 24 h post-transfection for downstream assays.

### RNA extraction and real-time quantitative PCR (RT-qPCR)

Total RNA was isolated using an RNA extraction kit (ESScience, RN001). cDNA synthesis was performed with HiScript II Reverse Transcriptase (Vazyme, RL201-01). RT-qPCR was conducted using ChamQ SYBR Master Mix (Vazyme, Q711-02) on a Bio-Rad CFX96 system. Primers are listed in Supplementary Table 2.

### Western blot and antibodies

Proteins were extracted using NP-40 lysis buffer (Beyotime, P0013F) supplemented with 1% PMSF (Beyotime, ST506). Protein concentrations were determined via the Bradford assay (Beyotime, P0006C). Equal amounts of protein (20–30 µg) were separated by 8% or 12% SDS-PAGE and transferred to PVDF membranes (Millipore, IPVH00010) using a Bio-Rad Trans-Blot system. Membranes were blocked with 5% non-fat milk in TBST for 1 h at room temperature and incubated overnight at 4°C with primary antibodies. After washing with TBST, membranes were probed with HRP-goat-anti-rabbit IgG (Proteintech, SA00001-2) or HRP-goat-anti-mouse IgG (Proteintech, SA00001-1) for 1 h at room temperature. Protein bands were visualized using an ECL detection kit (Thermo Scientific, 26625) and imaged on a Tanon chemiluminescence system.

The following primary antibodies were used by this research: anti-CPT1A (Abcam, ab220789; 1:1000); anti-β-actin (Proteintech, 66009-1-Ig; 1:20000); anti-Flag (Abclonal, AE005; 1:10000); anti-HA (Proteintech, 66006-2-Ig; 1:2000); anti-Histone H3 (PTM BIO, PTM-1001RM; 1:1000); anti-L-Lactyl-Histone H3 (K18) (PTM BIO, PTM-1427RM; 1:1000); anti-L-lactyl lysine (PTM BIO, PTM-1401RM; 1:1000).

### Dual-Luciferase reporter assay

Cells were co-transfected with firefly luciferase reporter plasmids (pCPT1A-Fluc-SV40-hRluc) and Renilla luciferase control vectors using Lipo8000 transfection reagent (Beyotime, C0533). After 48 h, cells were lysed with Passive Lysis Buffer (Promega, E1910), and lysates were centrifuged at 12,000×g for 10 min at 4 °C. Firefly and Renilla luciferase activities were sequentially measured using the Dual-Luciferase® Reporter Assay System (Promega, E1910) on a GloMax® Navigator luminometer. Firefly luciferase signals were normalized to Renilla luciferase values to account for variations in transfection efficiency. Data were analyzed using GraphPad Prism 8 and expressed as relative luciferase activity (firefly/Renilla ratio).

### Chromatin immunoprecipitation quantitative PCR (ChIP-qPCR)

A Pierce Magnetic ChIP Kit (Thermo Scientific Kit, 26157) was used according to the manufacturer’s procedure in our research. Chromatin was crosslinked with 1% formaldehyde, sonicated, and immunoprecipitated using anti-CPT1A. Precipitated DNA was analyzed by qPCR with primers targeting the CPT1A promoter (Supplementary Table 3).

### Co-Immunoprecipitation (Co-IP)

Cells were washed with PBS and lysed in ice-cold BC100 buffer supplemented with protease inhibitors. Lysates were centrifuged at 12,000×g for 10 min at 4 °C, and supernatants were quantified using the Bradford assay (Beyotime, P0006C). For each IP reaction, 1 mg of total protein was incubated with 25 μL Anti-Flag M2 magnetic beads (Sigma, M8823) overnight at 4 °C with gentle rotation. Beads were washed three times with BC100 buffer to remove nonspecific binding. Bound proteins were eluted in 2× Laemmli buffer containing 2% β-mercaptoethanol by boiling at 100 °C for 10 min. Input lysates (10% of total) and IP eluates were analyzed by Western blot using antibodies against Flag (Abclonal, AE005; 1:10000) or interacting partners.

### Subcutaneous tumorigenesis model

All animal experiments were approved by the Institutional Animal Care and Use Committee of Shandong University (Approval No. 23032). Male BALB/c-nude mice (5 weeks old) were purchased from GemPharmatech Co., Ltd. and acclimatized for 1 week under SPF conditions. Mice were randomly divided into four groups (*n* = 6 per group): (1) Vector control, (2) CPT1A-WT overexpression, (3) CPT1A-K180R mutant, and (4) CPT1A-K285R mutant. BHP10-3 cells stably overexpressing wild-type or mutant CPT1A (1×10⁷ cells in 100 μL PBS) were subcutaneously injected into the dorsal flank of each mouse. Tumor dimensions (length [L] and width [W]) were measured weekly using digital calipers, and tumor volume was calculated as (V = L×W^2^/2). Mice were euthanized at day 28 post-injection, and tumors were excised, weighed, and photographed.

### Tail vein-lung metastasis model

Male BALB/c-nude mice (5 weeks old) were acclimatized for 1 week under SPF conditions and randomized into four groups : (1) Vector control (*n* = 5), (2) CPT1A-WT overexpression (*n* = 5), (3) CPT1A-K180R mutant (*n* = 8), and (4) CPT1A-K285R mutant (*n* = 8). The investigators were not blinded to group allocation during the experiments and outcome assessment. BHP10-3 cells (2 × 10⁶ cells in 100 μL PBS) stably expressing CPT1A variants were injected into the lateral tail vein using a 29-gauge needle. A second injection with the same cell number was administered one week later to enhance metastatic potential. After 8 weeks, mice were euthanized, and lungs were harvested, fixed in 4% paraformaldehyde for 48 h, and paraffin-embedded. Serial sections (5 μm thickness) were stained with hematoxylin and eosin (H&E) for histological examination. Metastatic nodules were quantified by measuring total metastatic area using ImageJ software.

### Statistical analysis

Data analysis was performed using RStudio (version 4.0.2) for bioinformatics processing, with the “data. table” and “DESeq2” packages applied for data normalization and differential gene expression analysis. Statistical tests were conducted using GraphPad Prism 8.0. Data are expressed as mean ± standard deviation (SD) for normally distributed variables or median (interquartile range) for non-normally distributed variables. Group comparisons were analyzed using Student’s *t*-test or one-way ANOVA. Significance levels were defined as follows: ns (*p* ≥ 0.05), * (*p* < 0.05), ** (*p* < 0.01), and *** (*p* < 0.001). All experiments were independently repeated at least three times.

## Supplementary information

Supplementary Figure 1

Supplementary file

Checklist

Original western blots

supplementary figure 1 legend

## Data Availability

All data generated and analyzed during this study are included in this published article are available on request.

## References

[1] Bray F, Laversanne M, Sung H, Ferlay J, Siegel RL, Soerjomataram I, et al. Global cancer statistics 2022: GLOBOCAN estimates of incidence and mortality worldwide for 36 cancers in 185 countries. CA: A Cancer Journal for Clinicians. 2024;74:229–263. 38572751 10.3322/caac.21834

[2] LeClair K, Bell KJL, Furuya-Kanamori L, Doi SA, Francis DO, Davies L. Evaluation of gender inequity in thyroid cancer diagnosis. JAMA Intern Med. 2021;181. 10.1001/jamainternmed.2021.4804 PMC8406211 34459841

[3] Agrawal N, Akbani R, Aksoy BA, Ally A, Arachchi H, Asa Sylvia L, et al. Integrated genomic characterization of papillary thyroid carcinoma. Cell. 2014;159:676–690. 25417114 10.1016/j.cell.2014.09.050 PMC4243044

[4] Qu N, Chen D, Ma B, Zhang L, Wang Q, Wang Y, et al. Integrated proteogenomic and metabolomic characterization of papillary thyroid cancer with different recurrence risks. Nat Commun. 2024;15. 10.1038/s41467-024-47581-1 PMC11014849 38609408

[5] Papini E, Guglielmi R, Novizio R, Pontecorvi A, Durante C. Management of low-risk papillary thyroid cancer. Minimally-invasive treatments dictate a further paradigm shift? Endocrine. 2024;85:584–592. 38767774 10.1007/s12020-024-03864-7 PMC11291527

[6] Wang Q, Yu B, Zhang S, Wang D, Xiao Z, Meng H, et al. Papillary thyroid carcinoma: correlation between molecular and clinical features. Molecular Diagnosis & Therapy. 2024;28:601–609. 38896179 10.1007/s40291-024-00721-1 PMC11349796

[7] Lam AK-Y, Lo C-Y, Lam KS-L. Papillary carcinoma of thyroid: A 30-yr clinicopathological review of the histological variants. Endocrine Pathology. 2005;16:323–330. 16627919 10.1385/ep:16:4:323

[8] Tuttle RM, Haugen B, Perrier ND. Updated American Joint Committee on Cancer/Tumor-Node-Metastasis Staging System for Differentiated and Anaplastic Thyroid Cancer (Eighth Edition): What Changed and Why? Thyroid. 2017;27:751–756. 28463585 10.1089/thy.2017.0102 PMC5467103

[9] Vaupel P, Multhoff G. Revisiting the Warburg effect: historical dogma versus current understanding. The Journal of Physiology. 2021;599:1745–1757. 33347611 10.1113/JP278810

[10] Certo M, Llibre A, Lee W, Mauro C. Understanding lactate sensing and signalling. Trends in Endocrinology & Metabolism. 2022;33:722–735. 35999109 10.1016/j.tem.2022.07.004

[11] Gao Y, Zhou H, Liu G, Wu J, Yuan Y, Shang A, et al. Tumor microenvironment: lactic acid promotes tumor development. Journal of Immunology Research. 2022;2022:1–8. 10.1155/2022/3119375 PMC9207018 35733921

[12] Chen D, Liu P, Lu X, Li J, Qi D, Zang L, et al. Pan-cancer analysis implicates novel insights of lactate metabolism into immunotherapy response prediction and survival prognostication. J Exp Clin Cancer Res. 2024;43. 10.1186/s13046-024-03042-7 PMC11044366 38664705

[13] Hirschhaeuser F, Sattler UGA, Mueller-Klieser W. Lactate: a metabolic key player in cancer. Cancer Research. 2011;71:6921–6925. 22084445 10.1158/0008-5472.CAN-11-1457

[14] Yang L, Moss T, Mangala LS, Marini J, Zhao H, Wahlig S, et al. Metabolic shifts toward glutamine regulate tumor growth, invasion and bioenergetics in ovarian cancer. Mol Syst Biol. 2014;10. 10.1002/msb.20134892 PMC4188042 24799285

[15] Végran F, Boidot R, Michiels C, Sonveaux P, Feron O. Lactate influx through the endothelial cell monocarboxylate transporter MCT1 supports an NF-κB/IL-8 pathway that drives tumor angiogenesis. Cancer Research. 2011;71:2550–2560. 21300765 10.1158/0008-5472.CAN-10-2828

[16] Husain Z, Huang Y, Seth P, Sukhatme VP. Tumor-derived lactate modifies antitumor immune response: effect on myeloid-derived suppressor cells and NK cells. The Journal of Immunology. 2013;191:1486–1495. 23817426 10.4049/jimmunol.1202702

[17] Faubert B, Li KY, Cai L, Hensley CT, Kim J, Zacharias LG, et al. Lactate metabolism in human lung tumors. Cell. 2017;171:358–371.e359. 28985563 10.1016/j.cell.2017.09.019 PMC5684706

[18] Romero-Garcia S, Moreno-Altamirano MMB, Prado-Garcia H, Sánchez-García FJ. Lactate contribution to the tumor microenvironment: mechanisms, effects on immune cells and therapeutic relevance. Front Immunol*.* 2016;7. 10.3389/fimmu.2016.00052 PMC4754406 26909082

[19] Zhang D, Tang Z, Huang H, Zhou G, Cui C, Weng Y, et al. Metabolic regulation of gene expression by histone lactylation. Nature. 2019;574:575–580. 31645732 10.1038/s41586-019-1678-1 PMC6818755

[20] Yu J, Chai P, Xie M, Ge S, Ruan J, Fan X, et al. Histone lactylation drives oncogenesis by facilitating m6A reader protein YTHDF2 expression in ocular melanoma. Genome Biol. 2021;22. 10.1186/s13059-021-02308-z PMC7962360 33726814

[21] Wang N, Wang W, Wang X, Mang G, Chen J, Yan X, et al. Histone lactylation boosts reparative gene activation post–myocardial infarction. Circulation Research. 2022;131:893–908. 36268709 10.1161/CIRCRESAHA.122.320488

[22] Maschari D, Saxena G, Law TD, Walsh E, Campbell MC, Consitt LA. Lactate-induced lactylation in skeletal muscle is associated with insulin resistance in humans. Front Physiol*.* 2022;13. 10.3389/fphys.2022.951390 PMC9468271 36111162

[23] Pavlova Natalya N, Thompson Craig B. The emerging hallmarks of cancer metabolism. Cell Metabolism. 2016;23:27–47. 26771115 10.1016/j.cmet.2015.12.006 PMC4715268

[24] Ippolito L, Comito G, Parri M, Iozzo M, Duatti A, Virgilio F, et al. Lactate rewires lipid metabolism and sustains a metabolic–epigenetic axis in prostate cancer. Cancer Research. 2022;82:1267–1282. 35135811 10.1158/0008-5472.CAN-21-0914 PMC7612586

[25] Wolfgang MJ, Lane MD. The role of hypothalamic malonyl-CoA in energy homeostasis. Journal of Biological Chemistry. 2006;281:37265–37269. 17018521 10.1074/jbc.R600016200

[26] Samudio I, Harmancey R, Fiegl M, Kantarjian H, Konopleva M, Korchin B, et al. Pharmacologic inhibition of fatty acid oxidation sensitizes human leukemia cells to apoptosis induction. Journal of Clinical Investigation. 2010;120:142–156. 20038799 10.1172/JCI38942 PMC2799198

[27] Xiong Y, Liu Z, Zhao X, Ruan S, Zhang X, Wang S, et al. CPT1A regulates breast cancer-associated lymphangiogenesis via VEGF signaling. Biomedicine & Pharmacotherapy. 2018;106:1–7. 29940537 10.1016/j.biopha.2018.05.112

[28] Schlaepfer IR, Rider L, Rodrigues LU, Gijón MA, Pac CT, Romero L, et al. Lipid catabolism via CPT1 as a therapeutic target for prostate cancer. Molecular Cancer Therapeutics. 2014;13:2361–2371. 25122071 10.1158/1535-7163.MCT-14-0183 PMC4185227

[29] Lu J, Zhang Y, Sun M, Ding C, Zhang L, Kong Y, et al. Multi-omics analysis of fatty acid metabolism in thyroid carcinoma. Front Oncol*.* 2021;11. 10.3389/fonc.2021.737127 PMC8717782 34976793

[30] Yu X, Yang J, Xu J, Pan H, Wang W, Yu X, et al. Histone lactylation: from tumor lactate metabolism to epigenetic regulation. International Journal of Biological Sciences. 2024;20:1833–1854. 38481814 10.7150/ijbs.91492 PMC10929197

[31] Wang F-x, Mu G, Yu Z-h, Shi Z-a, Li X-x, Fan X, et al. Lactylation: a promising therapeutic target in ischemia-reperfusion injury management. Cell Death Discov. 2025;11. 10.1038/s41420-025-02381-4 PMC11906755 40082399

[32] Yang Z, Yan C, Ma J, Peng P, Ren X, Cai S, et al. Lactylome analysis suggests lactylation-dependent mechanisms of metabolic adaptation in hepatocellular carcinoma. Nature Metabolism. 2023;5:61–79. 36593272 10.1038/s42255-022-00710-w

[33] Srinivas SonneR, Gopal E, Zhuang L, Itagaki S, Martin PamelaM, Fei Y-J, et al. Cloning and functional identification of slc5a12 as a sodium-coupled low-affinity transporter for monocarboxylates (SMCT2). Biochemical Journal. 2005;392:655–664. 16104846 10.1042/BJ20050927 PMC1316307

[34] Halestrap AP, Wilson MC. The monocarboxylate transporter family—Role and regulation. IUBMB Life. 2011;64:109–119. 22162139 10.1002/iub.572

[35] Gatenby RA, Gillies RJ. Why do cancers have high aerobic glycolysis? Nature Reviews Cancer. 2004;4:891–899. 15516961 10.1038/nrc1478

[36] Lee YJ, Shin KJ, Park SA, Park KS, Park S, Heo K, et al. G-protein-coupled receptor 81 promotes a malignant phenotype in breast cancer through angiogenic factor secretion. Oncotarget. 2016;7:70898–70911. 27765922 10.18632/oncotarget.12286 PMC5342597

[37] Roland CL, Arumugam T, Deng D, Liu SH, Philip B, Gomez S, et al. Cell surface lactate receptor GPR81 is crucial for cancer cell survival. Cancer Research. 2014;74:5301–5310. 24928781 10.1158/0008-5472.CAN-14-0319 PMC4167222

[38] Feng J, Yang H, Zhang Y, Wei H, Zhu Z, Zhu B, et al. Tumor cell-derived lactate induces TAZ-dependent upregulation of PD-L1 through GPR81 in human lung cancer cells. Oncogene. 2017;36:5829–5839. 28604752 10.1038/onc.2017.188

[39] Ippolito L, Morandi A, Giannoni E, Chiarugi P. Lactate: a metabolic driver in the tumour landscape. Trends in Biochemical Sciences. 2019;44:153–166. 30473428 10.1016/j.tibs.2018.10.011

[40] Bonnefont JP, Demaugre F, Prip-Buus C, Saudubray JM, Brivet M, Abadi N, et al. Carnitine palmitoyltransferase deficiencies. Molecular Genetics and Metabolism. 1999;68:424–440. 10607472 10.1006/mgme.1999.2938

[41] Longo N, Amat di San Filippo C, Pasquali M. Disorders of carnitine transport and the carnitine cycle. American Journal of Medical Genetics Part C: Seminars in Medical Genetics. 2006;142C:77–85. 16602102 10.1002/ajmg.c.30087 PMC2557099

[42] Ma L, Chen C, Zhao C, Li T, Ma L, Jiang J, et al. Targeting carnitine palmitoyl transferase 1A (CPT1A) induces ferroptosis and synergizes with immunotherapy in lung cancer. Sign Transduc Target Ther. 2024;9. 10.1038/s41392-024-01772-w PMC10920667 38453925

[43] Nandi I, Ji L, Smith HW, Avizonis D, Papavasiliou V, Lavoie C, et al. Targeting fatty acid oxidation enhances response to HER2-targeted therapy. Nat Commun. 2024;15. 10.1038/s41467-024-50998-3 PMC11297952 39097623

[44] Zhu Y, Wang Y, Li Y, Li Z, Kong W, Zhao X, et al. Carnitine palmitoyltransferase 1A promotes mitochondrial fission by enhancing MFF succinylation in ovarian cancer. Commun. Biol*.* 2023;6. 10.1038/s42003-023-04993-x PMC10250469 37291333

[45] Jia MYX, Sun W, Zhou Q, Chang C, Gong W, Feng J, et al. ULK1-mediated metabolic reprogramming regulates Vps34 lipid kinase activity by its lactylation. Sci. Adv*.* 2023;9. 10.1126/sciadv.adg4993 PMC10413652 37267363

[46] Xie BZM, Li J, Cui J, Zhang P, Liu F, Wu Y, et al. KAT8-catalyzed lactylation promotes eEF1A2-mediated protein synthesis and colorectal carcinogenesis. Proc. Natl Acad. Sci*.* 2024, 121. 10.1073/pnas.2314128121 PMC10895275 38359291

[47] Ju J, Zhang H, Lin M, Yan Z, An L, Cao Z, et al. The alanyl-tRNA synthetase AARS1 moonlights as a lactyltransferase to promote YAP signaling in gastric cancer. J. Clin. Investig. 2024;134. 10.1172/JCI174587 PMC11093599 38512451

[48] Zong Z, Xie F, Wang S, Wu X, Zhang Z, Yang B, et al. Alanyl-tRNA synthetase, AARS1, is a lactate sensor and lactyltransferase that lactylates p53 and contributes to tumorigenesis. Cell. 2024;187:2375–2392.e2333. 38653238 10.1016/j.cell.2024.04.002

[49] Zhang D, Gao J, Zhu Z, Mao Q, Xu Z, Singh PK, et al. Lysine l-lactylation is the dominant lactylation isomer induced by glycolysis. Nature Chemical Biology. 2024;21:91–99. 39030363 10.1038/s41589-024-01680-8 PMC11666458

[50] Qiao Z, Li Y, Li S, Liu S, Cheng Y. Hypoxia-induced SHMT2 protein lactylation facilitates glycolysis and stemness of esophageal cancer cells. Molecular and Cellular Biochemistry. 2024;479:3063–3076. 38175377 10.1007/s11010-023-04913-x

[51] Zhang M, Zhao Y, Liu X, Ruan X, Wang P, Liu L, et al. Pseudogene MAPK6P4-encoded functional peptide promotes glioblastoma vasculogenic mimicry development. Commun. Biol. 2023;6. 10.1038/s42003-023-05438-1 PMC10584926 37853052

[52] Zhao Q. On the indirect relationship between protein dynamics and enzyme activity. Progress in Biophysics and Molecular Biology. 2017;125:52–60. 28163054 10.1016/j.pbiomolbio.2017.02.001

[53] Chen Y, Wu J, Zhai L, Zhang T, Yin H, Gao H, et al. Metabolic regulation of homologous recombination repair by MRE11 lactylation. Cell. 2024;187:294–311.e221. 38128537 10.1016/j.cell.2023.11.022 PMC11725302

[54] Wang J, Yang P, Yu T, Gao M, Liu D, Zhang J, et al. Lactylation of PKM2 Suppresses Inflammatory Metabolic Adaptation in Pro-inflammatory Macrophages. International Journal of Biological Sciences. 2022;18:6210–6225. 36439872 10.7150/ijbs.75434 PMC9682528

[55] Zhang R, Li L, Yu J. Lactate-induced IGF1R protein lactylation promotes proliferation and metabolic reprogramming of lung cancer cells. Open Life Sci. 2024;19. 10.1515/biol-2022-0874 PMC11151389 38840891

