# Supplementary figures and images for: Lactic acid promotes metastasis of papillary thyroid carcinoma by enhancing CPT1A lactylation
Source: Cell Death Dis. 2026 Apr 27;17(1):559. doi: 10.1038/s41419-026-08790-2 (PMC13254147; doi:10.1038/s41419-026-08790-2)

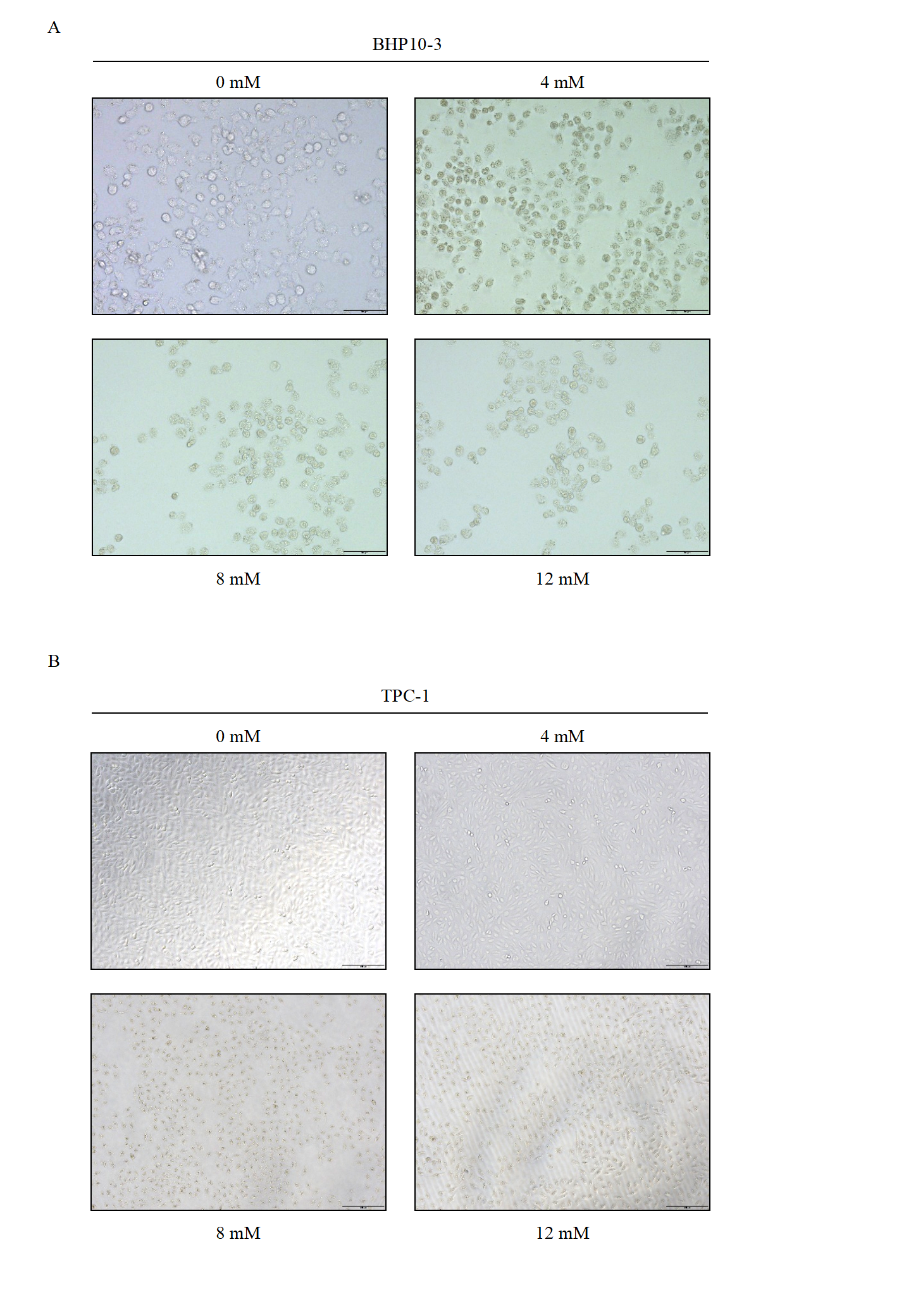

Supplement: Supplementary file 1 — Supplementary Figure 1 [file 41419_2026_8790_MOESM1_ESM.tif]

Figure 3A

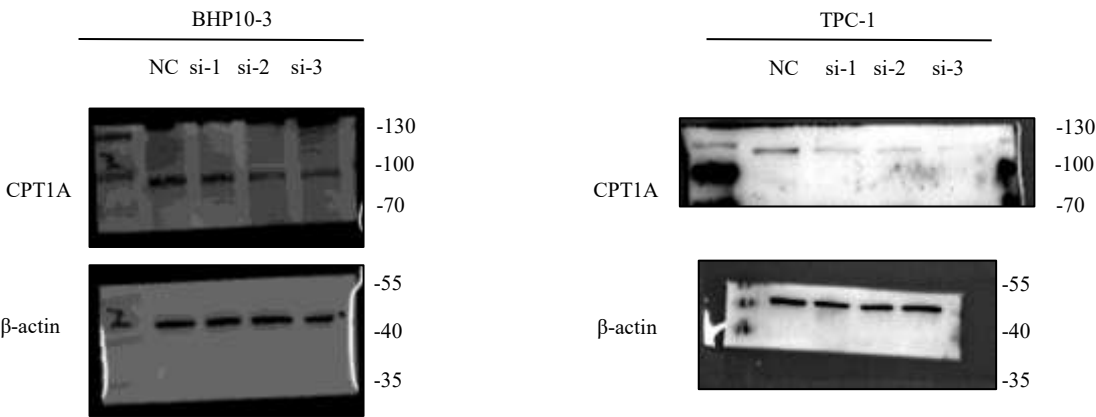

Figure 4D

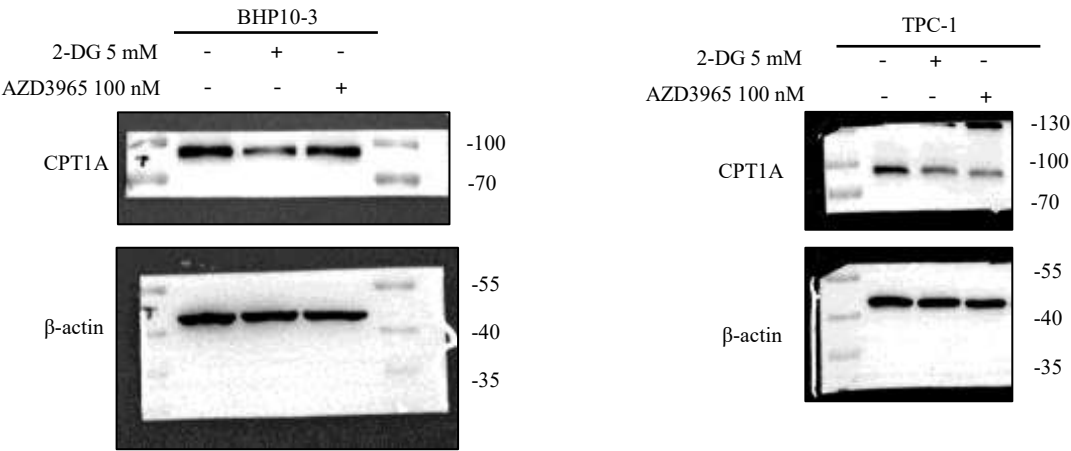

Figure 4G

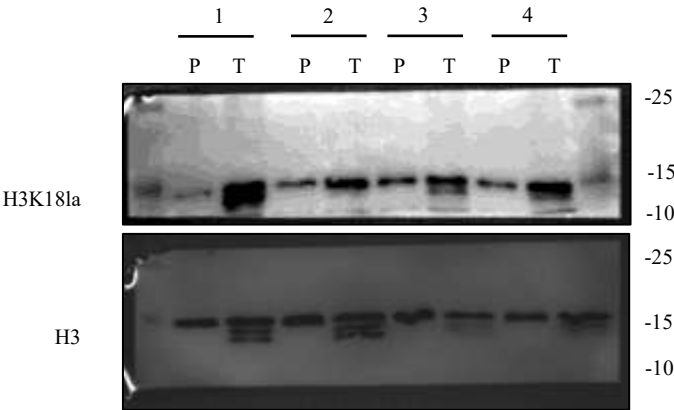

Figure 5A

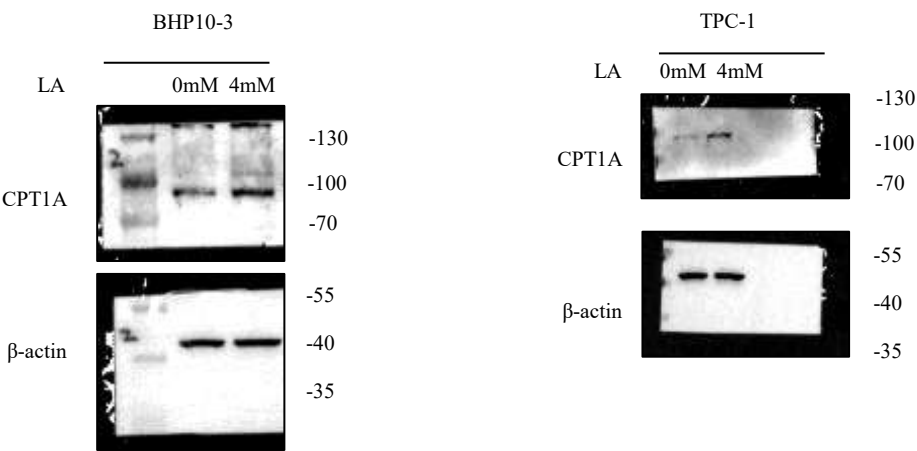

Figure 5B

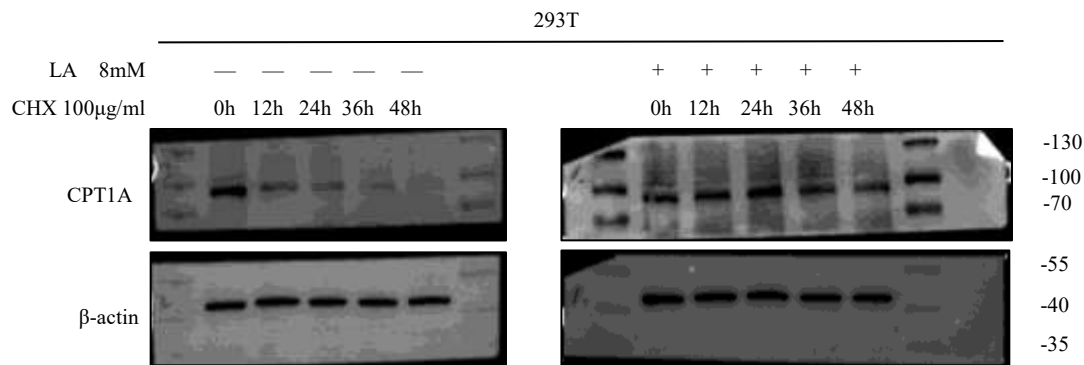

Figure 5D

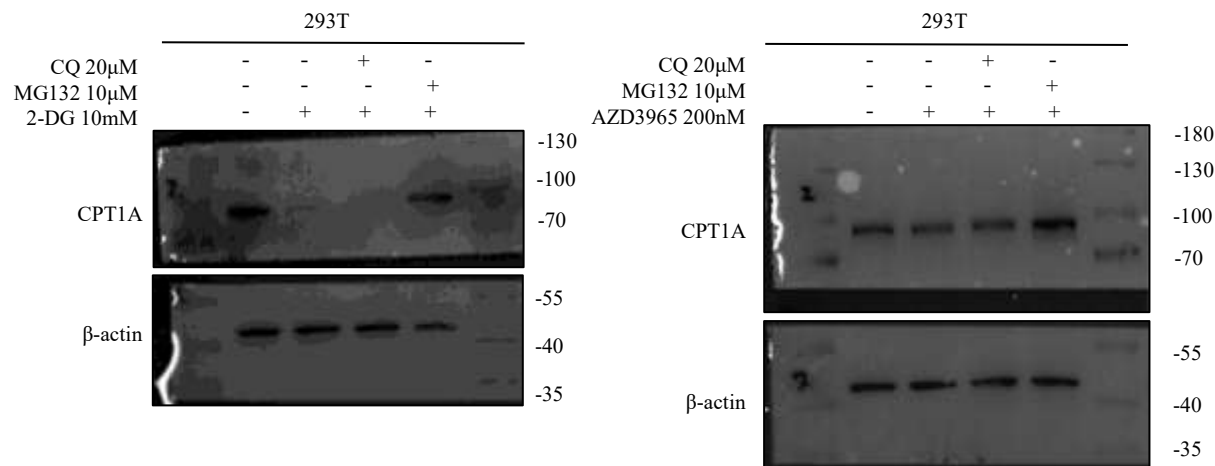

Figure 5E

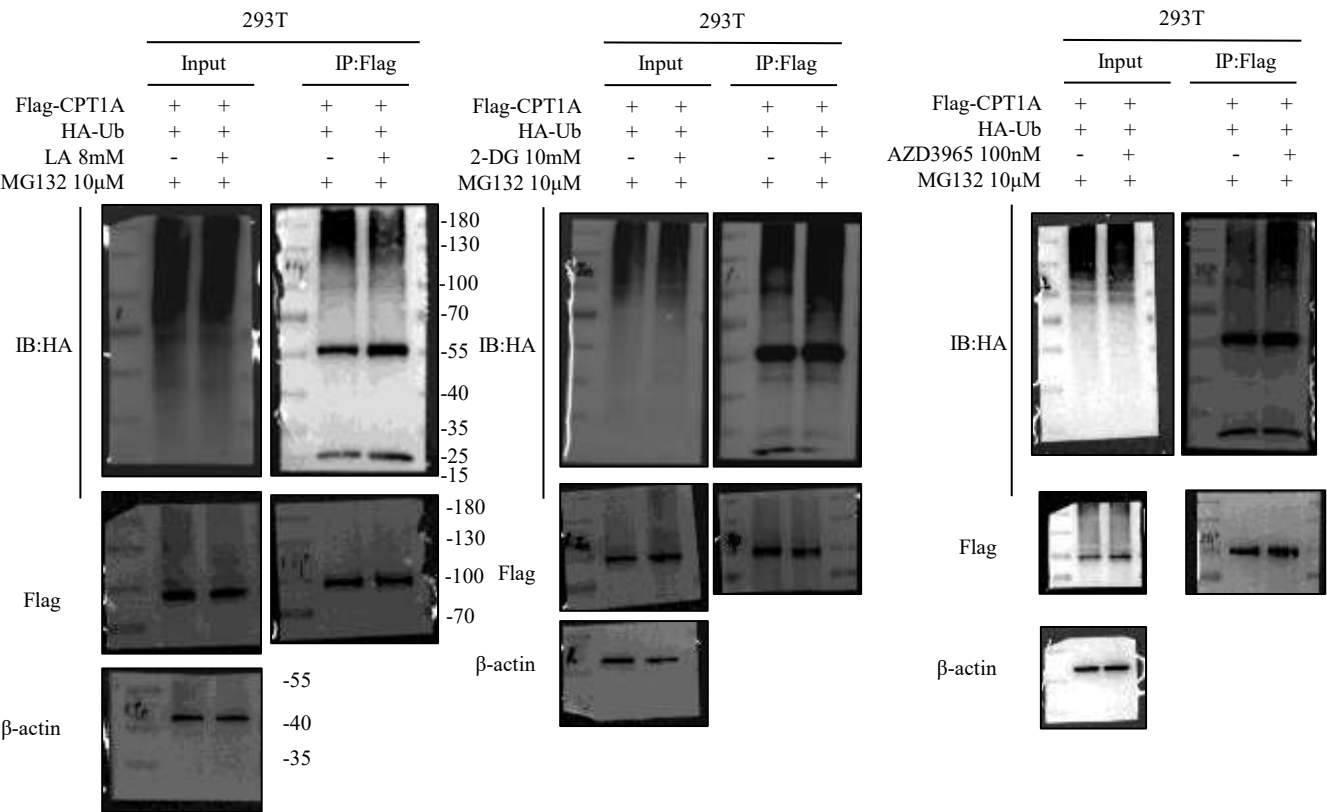

Figure 5G

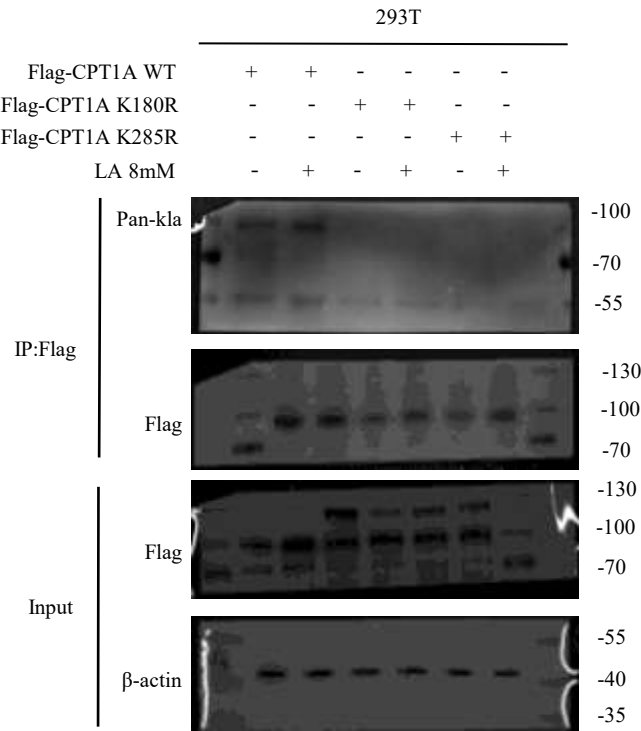

Figure 5H

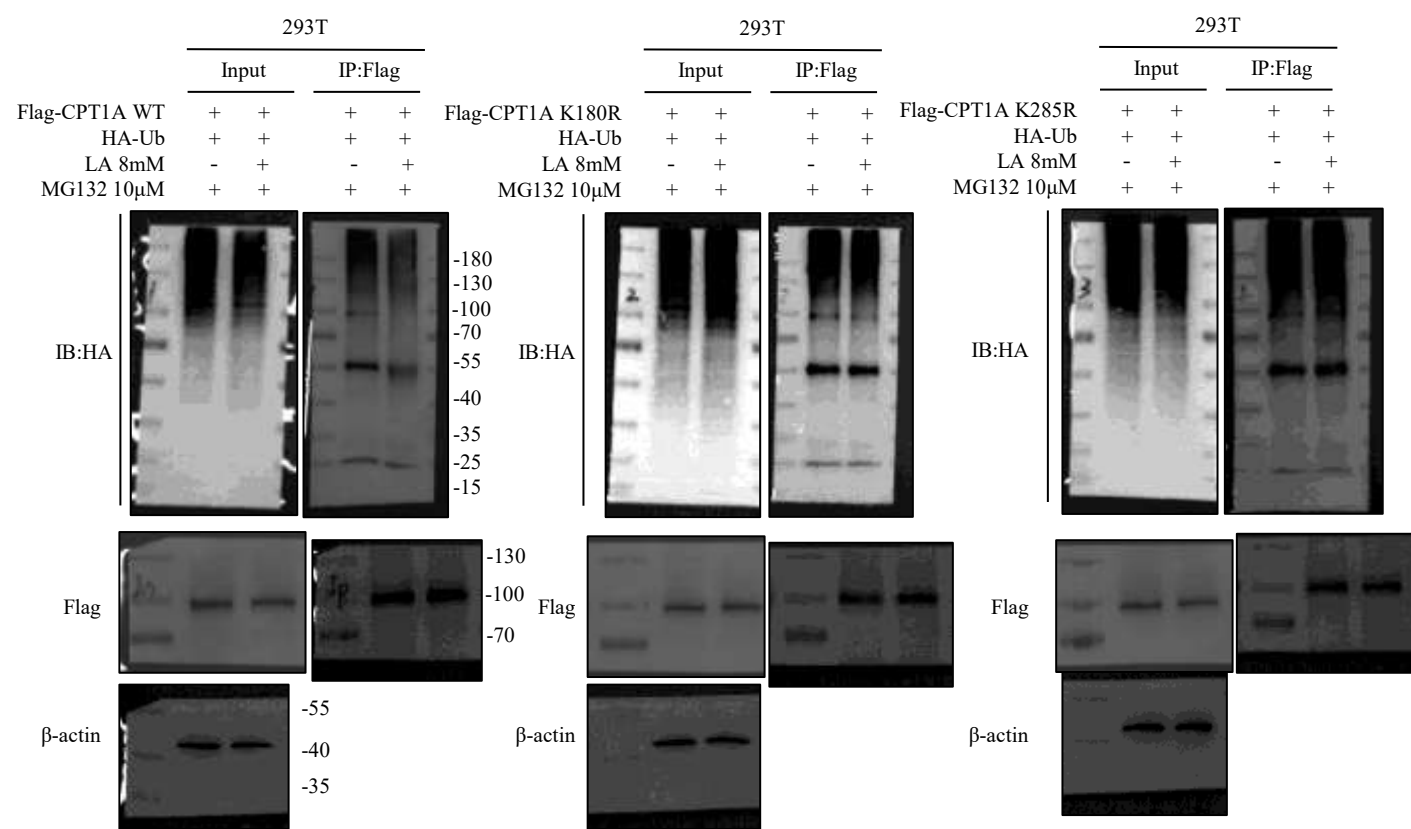

Figure 6A

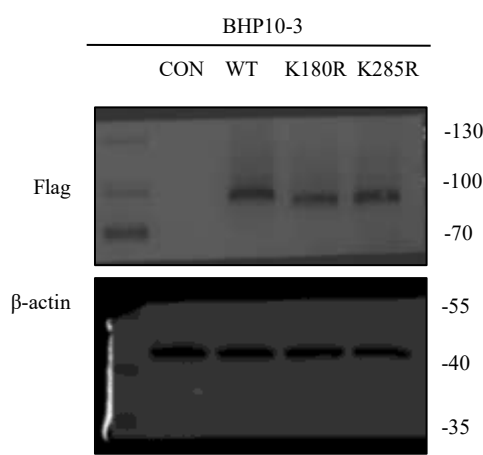

Supplement: Supplementary file 4 — Original western blots [file 41419_2026_8790_MOESM4_ESM.pdf]
