# Supplementary material for: Lactic acid promotes metastasis of papillary thyroid carcinoma by enhancing CPT1A lactylation
Source: Cell Death Dis. 2026 Apr 27;17(1):559. doi: 10.1038/s41419-026-08790-2 (PMC13254147; doi:10.1038/s41419-026-08790-2)
Supplement: Supplementary file 2 — Supplementary file [file 41419_2026_8790_MOESM2_ESM.docx]

**Supplementary table**

**Supplementary Table 1** siRNA sequence

| siRNA sequences | 5’-3’ |
| --- | --- |
| Srcamble-sense | UUCUCCGAACGUGUCACGUTT |
| Srcamble-antisense | ACGUGACACGUUCGGAGAATT |
| CPT1A (h)-siRNA-1-sense | CCGCAAAUCUUCUGGCAAATT |
| CPT1A (h)-siRNA-1-antisense | UUUGCCAGAAGAUUUGCGGTT |
| CPT1A (h)-siRNA-2-sense | CCAUGAAGCUCUUAGACAATT |
| CPT1A (h)-siRNA-2-antisense | UUGUCUAAGAGCUUCAUGGTT |
| CPT1A (h)-siRNA-3-sense | UGUUUGACUUGGAGAAUAATT |
| CPT1A (h)-siRNA-3-antisense | UUAUUCUCCAAGUCAAACATT |

**Supplementary Table 2** RT-qPCR primer sequences

| RT-qPCR primer sequences | 5’-3’ |
| --- | --- |
| β-actin (h)-F | TCCTGTGGCATCCACGAA |
| β-actin (h)-R | TCGTCATACTCCTGCTTGC |
| CPT1A (h)-F | TCCAGTTGGCTTATCGTGGTG |
| CPT1A (h)-R | TCCAGAGTCCGATTGATTTTTGC |

**Supplementary Table 3** ChIP-qPCR primer sequences

| ChIP-qPCR primer sequences | 5’-3’ |
| --- | --- |
| CPT1A (h)-promoter1-F | GCTTCTCAGCTCGGTGATGT |
| CPT1A (h)-promoter1-R | TATCTATCGGCATTGCCCCG |
| CPT1A (h)-promoter2-F | CTTGGCCCTCCTGTTACGC |
| CPT1A (h)-promoter2-R | GGGAGCGGGAACTGAACATC |
| CPT1A (h)-promoter3-F | CAGCCTTACAGACAGCTTGC |
| CPT1A (h)-promoter3-R | CGCTGCAGTTAACTCTCCCT |
