# Supplementary material for: Lactic acid promotes metastasis of papillary thyroid carcinoma by enhancing CPT1A lactylation
Source: Cell Death Dis. 2026 Apr 27;17(1):559. doi: 10.1038/s41419-026-08790-2 (PMC13254147; doi:10.1038/s41419-026-08790-2)
Supplement: Supplementary file 5 — supplementary figure 1 legend [file 41419_2026_8790_MOESM5_ESM.docx]

**Supplementary Fig. 1. Morphological changes of PTC cells treated with increasing concentrations of lactate.**

BHP10-3 cells (A) and TPC-1 cells (B) were treated with sodium L-lactate at the indicated concentrations (0, 4, 8, and 12 mM) for 24 hours. Cell morphology was observed and photographed under a light microscope. Concentrations of 8 mM and above induced noticeable cellular stress and morphological changes, including cell shrinkage and reduced adherence, whereas 4 mM lactate maintained normal cell morphology comparable to the untreated control (Scale bar: 100 μm).
